# Extracting quantitative information from single-molecule super-resolution imaging data with LAMA – LocAlization Microscopy Analyzer

**DOI:** 10.1038/srep34486

**Published:** 2016-10-05

**Authors:** Sebastian Malkusch, Mike Heilemann

**Affiliations:** 1 Single Molecule Biophysics, Institute of Physical and Theoretical Chemistry, Goethe-University Frankfurt, Germany

## Abstract

Super-resolution fluorescence microscopy revolutionizes cell biology research and provides novel insights on how proteins are organized at the nanoscale and in the cellular context. In order to extract a maximum of information, specialized tools for image analysis are necessary. Here, we introduce the LocAlization Microscopy Analyzer (LAMA), a comprehensive software tool that extracts quantitative information from single-molecule super-resolution imaging data. LAMA allows characterizing cellular structures by their size, shape, intensity, distribution, as well as the degree of colocalization with other structures. LAMA is freely available, platform-independent and designed to provide direct access to individual analysis of super-resolution data.

Super-resolution microscopy is about to expand the toolbox of microscopy techniques in many biological and biomedical research laboratories^1^,^2^. One approach for super-resolution microscopy is single-molecule localization microscopy (SMLM), where subsets of fluorophores are detected as single molecules and their positional information is used to reconstruct an image with subdiffraction spatial resolution^3^,^4^. SMLM allows visualizing cellular structures at a resolution of tens of nanometers and provides insight into nano-structural organization of proteins and their interaction^5^,^6^. Next to fluorescence images with superior spatial resolution, SMLM provides access to quantitative information at the single-molecule level, for example on protein copy numbers, stoichiometry, clustering and colocalization^7^. This additional and unique feature has its origin in the nature of SMLM raw data, i.e. precise information on the spatial position and time of detection of single fluorophore emission.

Software that generate single-molecule localization lists from SMLM data^8^ and for data-visualization^9^,^10^,^11^ are largely available. Recently, a software tool to simulate SMLM data from ground truth models was introduced^12^. Post-processing of SMLM data in order to extract biologically relevant and quantitative information is often performed individually, resulting in a portfolio of specialized algorithms^13^,^14^,^15^,^16^,^17^. A number of excellent post-processing software tools covering the various aspects of quantitative analysis of SMLM data is available^11^,^18^,^19^; such user-friendly and well-documented tools will make quantitative analysis of SMLM data accessible to non-expert users.

Here, we introduce *LocAlization Microscopy Analyzer* (LAMA), an open source, platform-independent software tool that solely concentrates on extracting quantitative information from SMLM data that is relevant for biological interpretation (Fig. 1). The input data for LAMA are lists of single-molecule localizations, as they are produced from common software packages for SMLM such as rapidSTORM^20^ or ThunderSTORM^10^. LAMA processes these localizations lists and uses a selection of algorithms to return information on the nanoscale organization of proteins.

A key benefit that super-resolution microscopy offers to quantitative cell biology is that the dimensions and the architecture of protein clusters can be analyzed with near-molecular spatial resolution. In the case of SMLM, this is not limited to intensity-based (pixel-based) algorithms commonly used for other super-resolution techniques, but allows for powerful coordinate-based algorithms. LAMA analyzes protein organization at a near-molecular scale utilizing algorithms for all-distance second-order statistics like Ripley’s K, L, and H function, as well as density-based data sorting into clustered regions (e.g. DBSCAN and OPTICS)^21^ (Fig. 1). In addition, we implemented a polygon-based morphology algorithm^22^ that extends DBSCAN and OPTICS and provides information on individual cluster size and copy numbers. LAMA also calculates the correlation of spatial patterns of two different proteins by using a coordinate-based colocalization algorithm^23^ (Fig. 1). In addition and as a measure for image quality, various approaches to estimate the localization precision are implemented: a selection of algorithms using photon statistics of individual molecules^24^,^25^, as well as an algorithm calculating a point-to-point distance distribution^26^ (Fig. 1). Fourier ring correlation^27^ was implemented and allows estimating the spatial resolution.

An ultimate goal in super-resolution data analysis is to determine protein complex stoichiometries. LAMA is the first software that allows extracting molecular stoichiometries by using an automated Markov-chain analysis of single-molecule photoswitching kinetics. With this feature, the oligomeric state of protein complexes can easily be determined from SMLM data^13^ (Fig. 1).

An exemplary workflow of LAMA will be illustrated with a two-color SMLM data set recorded for the HIV proteins gag and env in the plasma membrane of a T-cell (the data set is taken from^28^) (Fig. 2a). In a first step, the fiducial markers in the sample are used for accurate registration of multi-color SMLM images (Fig. 2b). In a second step, the quality of the SMLM image is determined by calculating the localization precision based on a point-to-point distance distribution^26^ (Fig. 2c). The localization precision is an important parameter that together with the labeling density determines the spatial resolution of an SMLM experiment^12^. In a next step the registered data is sorted into clustered regions (e.g. DBSCAN and OPTICS)^21^ (Fig. 2d). Further polygon-based morphology-analysis of the sorted data provides information on individual cluster size and copy numbers. In order to extract the population of interacting molecules the correlation of spatial patterns of two different proteins is calculated by using a coordinate-based colocalization (CBC) algorithm^23^ (Fig. 2e). Finally, the result of the CBC algorithm is combined with DBSCAN cluster analysis to extract domains of protein interactions (Fig. 2f).

LAMA was developed over several years in close cooperation with biology research groups along specific questions and is *a priori* designed to be easy to use for non-experts. LAMA introduces a simple data format that allows for combinations of different analysis algorithms and guarantees a high level of flexibility in experimental design. It comes with an extensive manual (available as supplemental information to this article), several tutorials and original data sets. Pre-compiled versions of LAMA (Windows, OSX) as well as its source code are available from our homepage (http://www.smb.uni-frankfurt.de).

## Additional Information

**How to cite this article**: Malkusch, S. and Heilemann, M. Extracting quantitative information from single-molecule super-resolution imaging data with LAMA – LocAlization Microscopy Analyzer. *Sci. Rep.* **6**, 34486; doi: 10.1038/srep34486 (2016).

## Supplementary Material

Supplementary Information

## Figures and Tables

**Figure 1:**
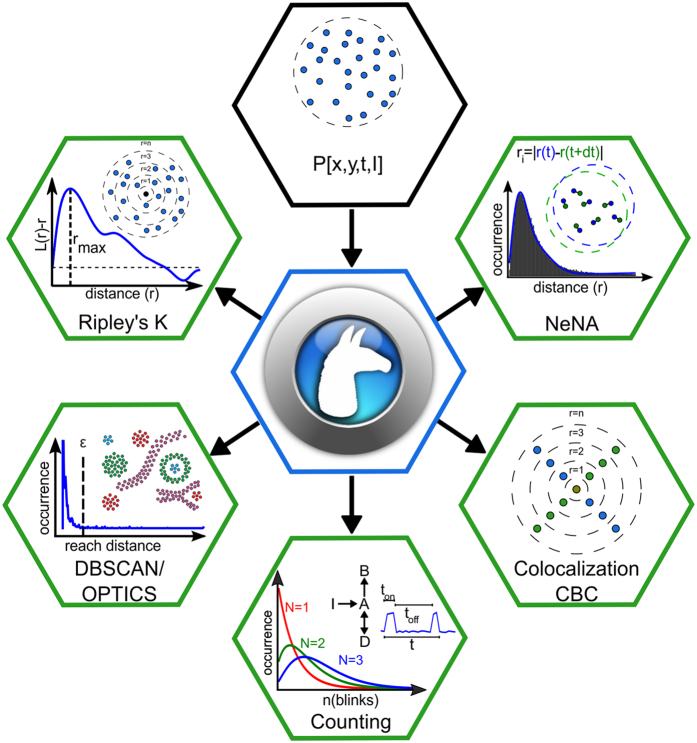
Quantitative analysis of SMLM data with LAMA. A list of single-molecule localizations serves as input data (top) for LAMA and is post-processed for clustering (Ripley functions, DBSCAN, OPTICS), single-molecule counting, colocalization analysis and localization precision determination.

**Figure 2:**
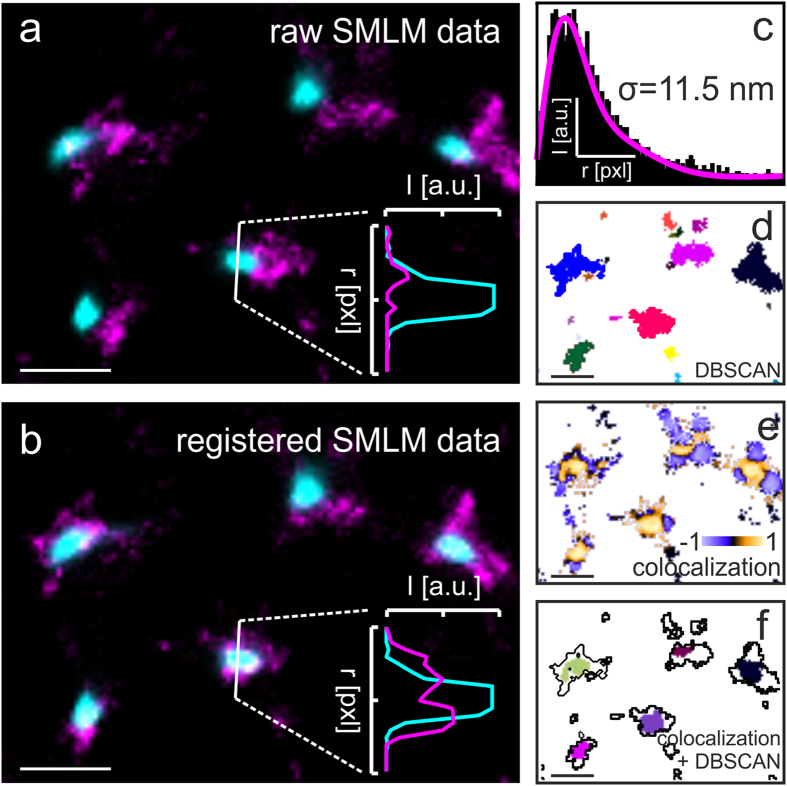
Quantitative analysis of SMLM data with LAMA. Exemplary two-color SMLM data set^8^ (**a**) before and (**b**) after color channel registration. LAMA includes algorithms for (**c**) determining the theoretical and experimental localization precision, (**d**) cluster analysis, (**e**) colocalization analysis and (**f**) a combination of colocalization and cluster analysis which reports on areas of molecular interaction (scale bars: 500 nm).
