# Supplementary material for: Extracting quantitative information from single-molecule super-resolution imaging data with LAMA – LocAlization Microscopy Analyzer
Source: Sci Rep. 2016 Oct 5;6:34486. doi: 10.1038/srep34486 (PMC5050494; doi:10.1038/srep34486)
Supplement: Supplementary Information [file srep34486-s1.pdf]

Extracting quantitative information from single-molecule super-resolution imaging data with LAMA – LocAlization Microscopy Analyzer

Sebastian Malkusch and Mike Heilemann

Single Molecule Biophysics, Institute of Physical and Theoretical Chemistry, Goethe-University Frankfurt, Germany

Correspondence: malkusch@chemie.uni-frankfurt.de, heilemann@chemie.uni-frankfurt.de

Supplementary information

Contents

1 Introduction 3
1.1 What is Lama . . . 3
1.2 What Lama can do . . . 4
1.3 What Lama cannot do . . . 4
1.4 Further analysis software . . . 4

2 Downloads 6

3 Installation 7
3.1 Install from binary file under Windows . . . 7
3.2 Install the app package under OSX . . . 7
3.3 Install the deb package under Ubuntu . . . 7
3.4 Installing Lama from source code . . . 7

4 The Philosophy of Lama 8
4.1 The input file format . . . 8
4.2 The graphical user interface . . . 8

5 The Main cabinet 9
5.1 The Input menu (Main cabinet) . . . 9
5.2 The ROI menu (Main cabinet) . . . 10
5.3 The Setup menu (Main cabinet) . . . 10

6 The Visualize cabinet 11
6.1 The Image menu (Visualize cabinet) . . . 12
6.2 The MCA menu (Visualize cabinet) . . . 12

7 The Precision cabinet 14
7.1 The Theoretical menu (Precision cabinet) . . . 14
7.2 The Experimental menu (Precision cabinet) . . . 16

8 The Fourier Ring Correlation cabinet 18

9 The Ripley's K-Function cabinet 19
9.1 Edge Correction in Lama . . . 20

10 The Register cabinet 21
10.1 The Bead Detection menu (Register cabinet) . . . 21
10.2 The Registration menu (Register cabinet) . . . 21

11 The CBC cabinet 22
11.1 CBC mediated cluster analysis . . . 25

12 The Hierarchical Clustering cabinet 25
12.1 The Sort menu (Hierarchical Clustering cabinet) . . . 25

13 The MCA menu (Hierarchical Clustering cabinet) 28

14 The stoichiometry cabinet 28
14.1 Probing the oligomeric state of reversibly photoswitching fluorophores . . . 29
14.2 Determining oligomeric states with Lama . . . 29

15 The Localization Converter 30

16 The Settings cabinet 30

Appendices 32

A Outlook 33
A.1 Ubuntu Linux support . . . 33
A.2 Drift Correction . . . 33
A.3 3D Data Sets . . . 33
A.4 Moving to c++ . . . 33

B Theory 34
B.1 The theory of NeNA . . . 34
B.2 Edge correction for Ripley's K-function . . . 36
Buffer Zones • Weighting Factor • Toroidal Edge Correction
B.3 On the use of Markov chains for the analysis of blinking kinetics at the single molecule level . . . 37

1. Introduction

Lama is a toolbox of algorithms that allows extracting quantitative information from *single-molecule localization microscopy* (SMLM) data. Lama is written in *python*, *cython* and *c*. The authors support the idea that research tools should be available to the community in a noncommercial way, which is why Lama is published under *GNU is Not Unix* (GNU) General Public License version 3 by the *Free Software Foundation*. We note that any localization microscopy software that generates lists of single-molecule localizations from raw image data can be used in combination with Lama. Among many other excellent tools, this for example includes ThunderSTORM [Ovesnỳ et al., 2014], rapidSTORM [Wolter et al., 2012], QuickPALM [Henriques et al., 2010], SimpleStorm [Köthe et al., 2014].

1.1 What is Lama

The actual success story of deterministic SMLM methods that circumvent the resolution limit defined by Abbe [Abbe, 1873], climaxed with the award of the 2014 Nobel Prize in chemistry to Eric Betzig, Stefan W. Hell and William E. Moerner [Stelzer, 2014]. These single-molecule super-resolution techniques are capable of localizing the fluorescence emission of single emitters with a precision of $\approx 10\,\mathrm{nm}$ (FIONA, PALM, STORM, dSTORM, GSDIM) [Selvin et al., 2007, Betzig et al., 2006, Rust et al., 2006, Heilemann et al., 2008, Fölling et al., 2008]. SMLM is performed on conventional wide-field fluorescence setups and relies on temporal confinement of fluorescence emission by detecting fluorophores individually, separating overlapping *point spread functions* (PSFs) in time. A key feature to realize this concept is the availability of photo-switchable fluorophores [Fürstenberg and Heilemann, 2013, van de Linde and Sauer, 2014]. Although different approaches of SMLM employ different photo-chemical or -physical effects to achieve appropriate photo-switching rates, their basic principle of determining the position of single fluorophores is the same:

- transfer all fluorophores to a non-fluorescent or spectrally different state
- stochastically activate a subset of fluorophores with a density low enough that the PSFs of single emitters do not overlap
- determine position and additional information on single fluorophores by analyzing the isolated PSFs (e.g. by a fitting procedure)
- repeat procedure until each fluorophore is detected at least once, or a statistically sufficient number of fluorophores was detected

To-date researchers can choose between several software packages performing different procedures to treat PSFs in SMLM image stacks. These software packages usually comprise two major algorithms: (i) a fast spot detection routine that identifies potential candidates as PSF of a single fluorophore; (ii) a more time consuming characterization of the possible hits that identifies single PSFs by their shape and intensity, which may group multiple detection events of the same single fluorophore. Among these software packages are *rapid yet accurate program implementing the direct stochastic optical reconstruction microscopy* (rapidSTORM), that uses a non-maximum suppression followed by a maximum likelihood estimation [Wolter et al., 2012], and WaveTracer, that uses a wavelet segmentation followed by a centroid estimation [Kechkar et al., 2013]. A wide range of SMLM software packages are compared and characterized at the Localization Microscopy Challenge [Sage et al., 2015]. The result of such

algorithms is a list that contains all the information extracted from an individually detected flourescent probe (e.g. spatial coordinates, time, intensity, etc.). These coordinate lists serve to reconstruct an artificial image of the sample, which provides sub-diffraction spatial resolution. In the simplest way, this is realized by binning all localization into a two-dimensional histogram.

The number of coordinates in a list naturally depends on the number of detected emitters, which in turn depends on the density at which a cellular structure was labeled with fluorophores, and the size of the field of view. In the following *Gedanken-experiment* the number of coordinates in the localization list shall be estimated for an ideal SMLM experiment. According to Deschout et al. [2014b] the quality of an SMLM experiment depends on the localization precision as well as on the labeling density. Therefore we assume an average localization precision of 10 nm and a labeling density of 1 label per 5 nm. For a homogeneously labeled 2D-structure within a field of view of 1 µm^2 and each fluorophore being detected only once, the localization list would contain 40,000 entries. In comparison, an image of this region recorded with a conventional wide field fluorescence setup and a *picture element* (pixel) size of 100 nm would contain 100 data points, which is exactly one per pixel. In conclusion SMLM experiments deliver much bigger data sets than comparable fluorescence microscopy experiments, that also differ significantly in their structure.

1.2 What Lama can do

Here, we introduce a software package that contains various algorithms recently developed and used for qualitative and quantitative data processing of SMLM experiments. The philosophy of Lama is based on the hypothesis, that the actual data that is received from a SMLM experiment is nothing more than a list of coordinates and that image formation is already the first step of data post processing. It therefore is mainly focussed on pure coordinate-based analysis of SMLM data, and for example provides information on localization precision, spatial localization distributions, cluster size, cluster composition, copy numbers, and colocalization.

1.3 What Lama cannot do

Lama is not a localization routine. It is developed for post-processing SMLM data and extraction of quantitative information. Lama requires a localization list as an input file. As it is focussed on coordinate based post-processing algorithms, it will provide nothing more than numbers. Lama will not generate beautiful images for publications. For visualization we recommend you to use your localization software of choice or to one of the post processing software packages specialized on visualization [El Beheiry and Dahan, 2013, Pengo et al., 2014].

1.4 Further analysis software

We also would like to highlight a number of other software tools, developed to analyze SMLM data quantitatively. Packages covering algorithms for multiple tasks for example include PALMSIEVER [Pengo et al., 2014], SharpVisu [Andronov et al., 2016a], and MIiSR [Caetano et al., 2015]; a huge and well-documented selection of algorithms for quantitative SMLM analysis is also provided by the Genome Damage and Stability Centre at the University of Sussex [Damage and at the University of Sussex]. A large selection of more specific algorithms has become available in the recent years [Deschout et al., 2014a]; many of the recent developments focus on how single-molecule localizations can be grouped into clusters and provide information on protein organization [Andronov et al., 2016b, Barsic et al., 2014, Levet et al., 2015]. Other approaches aim to extract information on protein copy

numbers and stoichiometry [Avilov et al., 2014, Fricke et al., 2015, Lee et al., 2012].

2. Downloads

In order to work with Lama you will need to install the following additional tools.
For a quick start you will need two software packages to be installed:

- A software that generates lists of single-molecule localizations from raw image data
- The Lama source code (python, cython, c) as well as pre-compiled versions for Mac OSX and Windows are freely available from the *Single Molecule Biophysics* (SMB) web page: www.smb.uni-frankfurt.de

For building Lama from the source code you will need the following packages to be installed.

- Python v2.7.10 from https://www.python.org
- setuptools v17.11 from https://pypi.python.org/pypi/setuptools
- numpy v1.9.2+mkl from http://www.numpy.org
- pillow v2.9.0 from https://pypi.python.org/pypi/Pillow/2.9.0
- matplotlib v1.4.3 from https://pypi.python.org/pypi/matplotlib/1.4.3
- scipy v0.14.0 from http://www.scipy.org
- openCV v3.0 fromhttp://opencv.org/opencv-3-0.html
- PyQt4 v4.11.4 from https://pypi.python.org/pypi/PyQt4/4.11.4
- cython from https://pypi.python.org/pypi/Cython/0.23.1
- a c++ compiler (gcc on Linux or clang on MacOSX or MS visual C++ compiler for Python 2.7)

In order to visualize images, you will need an image processing tool. We recommend one from the following list:

- ImageJ from http://rsb.info.nih.gov/ij/
- FIJI from http://fiji.sc/Fiji
- ICY from http://icy.bioimageanalysis.org

For further data processing and calculating statistics you will also need a spreadsheet program. As most of these are not open source we retain from recommending a specific software here.

3. Installation

This section describes the installation of Lama. A more detailed manual on the installation can be obtained from the SMB webpage (`http://www.smb.uni-frankfurt.de`).

3.1 Install from binary file under Windows

- uninstall any outdated version of Lama installed on your system!
- download the pre-compiled .msi installer of the latest version from SMB homepage
- execute the .msi installer
- start the programm by executing Lama.exe

3.2 Install the app package under OSX

- download the pre-compiled .app package from SMB homepage
- Lama must be executed from terminal!
- start the terminal
- switch to the Lama directory
- change directory by typing
  cd Lama-16.08.app/Contents/MacOS/
- start the program from the terminal by typing ./main

3.3 Install the deb package under Ubuntu

There are actually no stable pre-compiled rpm (openSUSE) or deb (UBUNTU) packages of Lama available. Therefore, if you are using Linux, you will have to run the program via the python interpreter. Please refer to the next section *Installing Lama from source*. A pre-compiled version for Ubuntu and openSUSE is planned in a future release.

3.4 Installing Lama from source code

The main program of Lama is written in python. Therefore you will need to have python and some additional packages installed on your system. We tested Lama successfully with python 2.7 and python 3.4. All necessary packages are listed in the *Downloads* section. As Lama is partly written in cython and c you will have to compile parts of the code. Therefore you will need to have a C++ compiler to be installed on your system (gcc on Linux, clang on MacOSX, or MS visual C++ compiler for Python 2.7 on Windows). In order to build Lama from source code follow the instructions below:

- install all packages from the *Downloads* section on your system
- download the Lama source code from the homepage
- extract the source code to a new folder
- start the terminal (or cmd on windows)
- go to the Lama source code directory
- execute the respective make-xxx.yy file for your system

Afterwards you may start Lama from within the source code directory by typing:
python2.7 Lama.py or *python3.4 Lama.py*.

4. The Philosophy of Lama

All data processing routines in Lama consist of two consecutive steps. First, the localization file is filtered based on user defined specifications and saved in a separate file. Second, the filtered data subset is passed to the desired processing algorithm (see algorithm 1). This way several sub-populations from a *single molecule* (SM) data set can be analyzed individually and compared later.

Algorithm 1: Lama data analysis engine

Data: single molecule localization list L;
Input: pre-sorting parameters S_{ROI};
Result: single molecule localization list L_{ROI};
Result: algorithm specific result file;
create empty list of localizations L_{ROI};
load single molecule localization list L;
for *all single molecule localizations I in L* **do**
    compare I with S_{ROI};
    if I *fits* S_{ROI} **then**
        write I to L_{ROI};
save L_{ROI};
start analysis algorithm with L_{ROI};
save specific result file;

Next to standard image-based analysis routines Lama comprises several coordinate-based algorithms that were adopted from other scientific fields or developed exclusively for SMLM during the last decade. At the current state of development Lama includes:

- visualization of super-resolution images [Malkusch et al., 2013]
- analysis of molecular subpopulations [Fricke et al., 2014]
- determining the localization precision [Thompson et al., 2002, Mortensen et al., 2010, Endesfelder et al., 2014, Rieger and Stallinga, 2014]
- determining the resolution of an SMLM image [Nieuwenhuizen et al., 2013]
- registration of multiple channels by automatic detection of fiducial markers [Zessin et al., 2013]
- calculation of the degree of clustering by Ripley's K-function [Ripley, 1976, Owen et al., 2010, Endesfelder et al., 2011]
- morphological cluster analysis [Muranyi et al., 2013]
- coordinate-based colocalization [Malkusch et al., 2012]
- sorting molecules into cluster by DBSCAN [Ester et al., 1996]
- obtaining most probable starting parameters for DBSCAN by OPTICS [Ankerst et al., 1999]
- single molecule counting [Fricke et al., 2015]

4.1 The input file format

For the sake of compatibility Lama introduces a minimalistic format of the localization lists as input and output: the *Molecular Accuracy Localization Keep* (Malk), comprising information that is available from nearly every SMLM software (see table 1). The format reduces the collected information for every localization to 4 observables: the position in x-dimension, the position in y-dimension, the time of the detection event, and the integrated signal density of the *point spread function* (PSF). The Malk format is supported by rapidSTORM [Wolter et al., 2012]. In order to analyze data sets with different formats Lama comes with a file converter.

4.2 The graphical user interface

The *graphical user interface* (GUI) of Lama enables for intuitive data analysis and allows the user to optimize all parameters. The Lama GUI is organized as a filing cabinet menu making it easy to navigate between different analysis

Table 1. The Malk file format
Data structure of the *Molecular Accuracy Localization Keep* as used by Lama.

x [nm]	y [nm]	t [frame]	I [A.D. counts]
4.61279e+03	1.60839e+04	1	1.23263e+05
9.46230e+03	6.86257e+03	2	2.23775e+04
8.02934e+03	7.41184e+03	2	7.45975e+03
6.90199e+03	1.33447e+04	3	8.71320e+03
...	...	...	...

routines (see figure 1). For reproducibility reasons Lama saves the current state of settings to a separate file. These files may later be used for batch analysis of multiple SMLM files (in particular useful for analyzing large data sets). The main window of Lama consists of an input menu that takes pre-sorting parameters and a drop-down button menu to navigate between the different analysis functions.

5. The Main cabinet

After starting Lama the *Main* cabinet shows up. The *Main* cabinet comprises three menus: the *Input* menu, the *region of interest* (ROI) menu and the *Setup* menu.

5.1 The Input menu (Main cabinet)

Within the *Input* menu one can choose whether to analyze a single SMLM file, or a batch of multiple files. In the first case, a mouse-click on the *Browse* button opens an explorer to select the file to be analyzed (the file needs to be a .txt file in Malk format). For analyzing a single Malk file the *single localization file* radio button must be activated.

Analyzing many files at once is realized by activating the batch mode.

To do so you have to create a list.txt file comprising the paths to all the localization lists you would like to analyze (see table 2). Mouse-click on the *Browse* button and select the list comprising the paths and names of all files you want to analyze. Make sure that the *multiple localization file* radio button is activated. Lama will now loop through all listed files and analyze one by one with the same parameters (see algorithm 2).

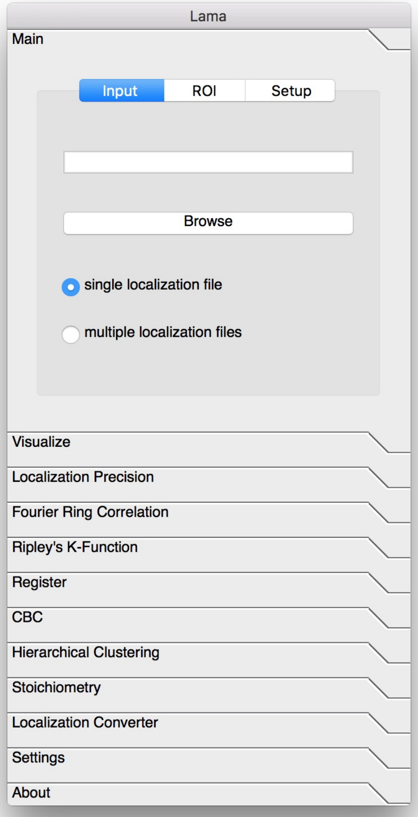

Figure 1. Lama Input menu (Main cabinet)
For analysis of a single SMLM experiment the path to the localization data file is chosen by mouse click on the *Browse* button and activated *single localization file* radio button. For batch analysis of multiple SMLM experiments the path to a text file containing a list of localization file paths is chosen by mouse click on the *Browse* button and the *multiple localization files* radio button has to be activated.

Table 2. The batch file format
Data structure of batch file that points to several Malk files to be analyzed by Lama.

batchfile.txt
c://folder01/folder02/folder.../filename01.txt
c://folder01/folder02/folder.../filename02.txt
c://folder01/folder02/folder.../filename...txt

Algorithm 2: Lama batch analysis engine

Data: single molecule localization file list L;
Result: single molecule localization lists L_{ROI};
Result: algorithm specific result files;
for *all SMLM localization files I in L* **do**
  process algorithm 1;

5.2 The ROI menu (Main cabinet)

After the input file(s) have been chosen, the filtering parameters need to be defined, as Lama will pre-filter the data before starting with its actual data processing routine (see figure 2). A ROI is defined as a subset of the initial data by defining 4 different filter parameters: the spatial parameters x and y define a field of view and will filter localizations that are placed outside this field; the time parameter t; the intensity parameter *int* filters the localization list by the integrated intensity of each localization. Those 4 parameters typically pre-filter the data set. The *CBC* filter parameter is induced for applications discussed in section 11.

The pre-filtering routine allows the analysis of different subpopulations within the same data set, like different fields of view or different time intervals. For example, if the localization list contains SM-data of two biological cells, pre-filtering would allow to address both cells individually. If the laser intensity has been changed during the measurement the time filter would allow to analyze each part of the experiment separately. The *Auto detect* button ensures that each localization from the data set is considered. The auto detect method is a high-low signal selector that automatically sets the parameters of the ROI.

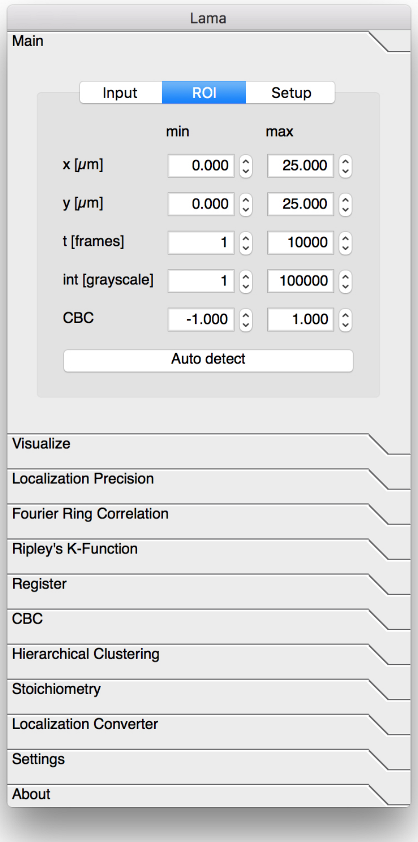

Figure 2. Lama ROI menu (Main cabinet)
Choosing different filtering parameters for the subpopulation definition.

5.3 The Setup menu (Main cabinet)

In the *Setup* menu of the *Main* cabinet the experimental conditions of the SMLM experiments are defined (see figure 3). One of them is the *grayscale conversion factor into photons*. It calculates photon numbers from the grayscale units of an image. The factor depends on the camera and recording software. The necessary information to calculate it is available in the instrument fact sheet. For many cameras used in the context of SMLM, the conversion factor is calculated from the electron multiplying gain (EM gain) and the *analog-to-digital* (ad) conversion factor (which depends on the pre-gain).

$$\text{GrayscaleConversionFactor} = \frac{\text{EMgain}}{\text{ADconversion}} \qquad (1)$$

For example, an ad conversion factor of 66 in combination with an EM gain of 200 would result in a *grayscale conver-*

sion factor into photons of about 3. If otherwise the integrated intensity is given in photons, the *grayscale conversion factor into photons* parameter is set to 1. The *setup pixel size* parameter contains the information on the edge length of a pixel from the diffraction limited raw image of the optical setup.

The *integration time per frame* is needed to calculate the exact time that a fluorescent probe was detected.

Not all parameters are needed by all data analysis routines of Lama. Respective sections in the manuals and the documentation indicate the input parameters required.

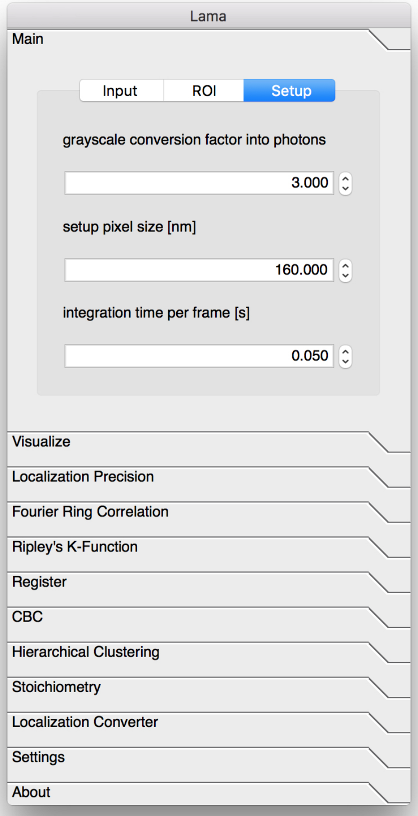

Figure 3. Lama Setup menu (Main cabinet)
Characterize the experimental setup conditions of the analyzed SMLM experiment.

6. The Visualize cabinet

Much software to visualize SMLM data is already available and Lama does not compete with these. In order to compute a nice image for a publication, the authors refer to the visualization output of other localization software (a recent overview can be found here [Sage et al., 2015]) or to use a software package specialized on visualizing SMLM data [El Beheiry and Dahan, 2013, Pengo et al., 2014].

The philosophy of Lama is that the raw result of an SMLM experiment is represented by the localization list. Visualization routines already represent a first step of data analysis. These routines are using some kind of imaging function or algorithm and convolve the localization list with this particular function in order to compute an image. This routine is somehow similar to the way a conventional fluorescence microscopic image is created, where the coordinates of all fluorescent probes (which act as point emitters) are convolved with the optical transfer function of the microscope. Therefore, SMLM images generated by different software do not necessary deliver comparable data. For example, if you calculate a cluster size of a protein and use an *SMLM* image that was created by a 2D histogram (where each localization is binned into an area of 4 pixels with a weighted intensity) and compare it to a second image computed by a Delaunay triangulation, you most likely end up with two different results.

So why implementing a *Visualization* cabinet anyway? One reason is to actually see wether your choice of pre-filter parameters is suitable for further analysis of your data. As most of localization files comprise up to millions of localizations, the coordinate-based routines of Lama may take some time. The visualization process is pretty quick and gives you a fast overview of your ROI. Another reason for the implementation of 2D-histogram function is the possibility to introduce

a quick cluster *Morphological Cluster Analysis* (MCA) that uses the morphology of cohesive regions on the one hand and the intensity information from the localization file on the other.

6.1 The Image menu (Visualize cabinet)

The coordinates of SMLM data are numbers with a certain localization error. The pixel size of a super resolved image can be chosen freely. This is done by typing an integer into the *desired pixel size* field. A mouse-click on the *Compute* button will produce two files: A localization file that comprises the filtered localizations and an image of the ROI. Re-defining the pre-filter parameters and repeating the *Visualization* will overwrite the results with the updated files.

The routine calculates a 2D-histogram of the localization list. The bin-size of the histogram is given by the *desired pixel size* parameter in both dimensions (Lama does not support non-squared bins). The image is saved in 8-bit or 256 gray scale values (0-255). The intensity of the pixel encode for the number of localizations binned within the area of the pixel. By default, the pixel containing the highest number of localizations is set to an intensity value of 255. The intensity of all other pixels is interpolated linearly with respect to the number of localizations.

A drawback of this setting is shown by the following scenario: In a very heterogeneous local distribution of localizations, areas with a low localization density may not be visualized. This will especially occur if bright and photo-stable fiducial markers are used, because these are detected in every frame. For example, lets consider an SMLM experiment of an ROI comprising 5 fiducial markers that are imaged over 10,000 frames. Afterwards, Lama is used to compute an image with a pixel size of 25 nm. In the worst case all localizations for each of the 5 fiducial markers will end up in a single pixel containing 10,000 localizations. The gray scale value of these 5 pixels will be set to 255 by Lama with the result that at least a population of 40 localization within a single pixel is needed to gain a gray scale value increase of 1.

This problem can be overcome by activating the *choose maximum manually* checkbox. This allows to choose the population value of a reference pixel manually. By default the value is set to 255.

Going back to the example this means the routine will now increase a pixel's gray scale value with respect to the number of localizations binned to this particular pixel. The output image reports the number of localizations per pixel.

Be aware that activating the *choose maximum manually* checkbox will give false information about the 5 pixels containing the fiducial marker localizations. As every localization above the maximum is not considered, they will only yield a value of 255.

Lama is also capable of blurring the images, such that structures may appear connected, by activating the *enable convolution* checkbox. The image will be calculated as described before, but additionally a second image is calculated that is convolved with a Gaussian low-pass with a user-defined root mean square deviation (default is $\sigma = 10\,\text{nm}$). This image is further referred to as *local weight matrix* (lwm)

6.2 The MCA menu (Visualize cabinet)

A quick and straight forward method to analyze protein clusters is available with MCA (see figure 5). The algorithm uses either the 2D-histogram or the lwm and an intensity threshold parameter to calculate a *binary cluster map* (bcm) (see algorithm 3).

MCA is an image-based way to analyze the clustering of proteins. The reconstructed image, whether it is blurred or not, is

Algorithm 3: Lama MCA analysis algorithm

Data: single molecule localization file list L_{ROI};
Input: optional: gaussian low-pass filter F_{lp};
Input: intensity threshold thr;
Input: list of filter parameter for cohesive region parameters F_{reg};
Result: 8-bit image of 2D-histogram I_{raw};
Result: optional: 8-bit image of a local weight matrix I_{lwm};
Result: binary image of cohesive regions I_{bcm};
Result: list of cohesive regions$L_{cluster}$;
receive L_{ROI} from Algorithm 1;
create empty list cohesive regions $L_{cluster}$;
create 2D-hitogram I_{raw} of L_{ROI};
save I_{raw};
if *Convolution is enabled* **then**
    create local weight matrix I_{lwm} by $I_{raw} * F_{lp}$;
    save I_{lwm};
    create binary image l_{bcm} by applying thr to I_{lwm};
else
    create binary image l_{bcm} by applying thr to I_{raw};
save I_{bcm};
detect cohesive regions from I_{bcm};
write region characteristics (center of mass, shape) from I_{bcm} to $L_{cluster}$;;
write region characteristics (number of localizations within shape) from I_{raw} to $L_{cluster}$;;
filter $L_{cluster}$ by F_{reg};
save $L_{cluster}$;

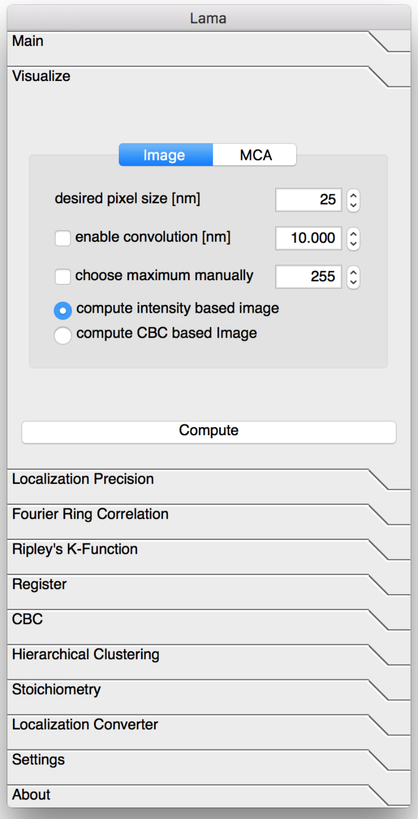

Figure 4. Lama Image menu (Visualize cabinet)
Visualize SMLM data by a 2D localization histogram. Choose the desired pixel size, and a population maximum; enable convolution with a Gaussian low-pass.

used to calculate a binary mask of the intensity distribution using an *intensity threshold* defined by the user. As SMLM data consist of absolute numbers in intensity with small errors in the localization precision, the morphological cluster analysis routine resigns further morphological image processing as erosion or dilation, as they could distort the image. The obtained binary masks of the clusters are further analyzed for their center of mass, size, and the radius of a coextensive circle. The list of detected clusters is additionally filtered by size based on the two extrem values *minimum cluster radius* and *maximum cluster radius* chosen by the user. So far the morphological cluster analysis routine equals conventional

image-based cluster algorithms like the *objects counter* plugin in ImageJ [Bolte and Cordelieres, 2006]. Its strength lies in the possibility of analyzing the number of localizations within each cluster in a quantitative way, by referring to the original localization list. The results are written into an additional .txt file (see table 3).

The *morphological cluster analysis* routine was tested on the viral Gag polyprotein of *human immunodeficiency virus* (HIV)-1 budding sites, as they are well defined in size by electron microscopy and tomography [Briggs et al., 2009, Malkusch et al., 2013]. It was also applied to investigate the clustering of *Env* glycoproteins [Muranyi et al., 2013], as well as arrestin2 proteins [Truan et al., 2013] in the cell membrane. The fact making the MCA analysis superior to pure image-based cluster analysis, is that the number of localizations is determined by combining image-based information as well as single molecule localization list information.

Table 3. The MCA file format
Data structure of the *MCA* result file created by Lama.

x [nm]	y [nm]	size [nm^2]	r_{coex} [nm]	I [localizations]
2.1449e+04	1.5019e+03	5.0000e+03	3.9894e+01	9.7e+01
1.5217e+04	1.5178e+03	1.8750e+03	2.4430e+01	5.2e+01
2.4922e+04	1.5436e+03	6.8750e+03	4.6780e+01	7.0e+00
1.0750e+03	1.5750e+03	6.2500e+02	1.4104e+01	5.9e+01
...	...	...	...	...

This method works well with densely clustered localizations that are characterized by defined constraints. However, if the structure of interest exhibits clusters of low and inhomogeneous density, MCA may be inappropriate. In this case Lama offers the use of Ripley's K-function (see chapter 9) or hierarchical clustering (see chapter 12).

7. The Precision cabinet

Lama offers in principle two ways of determining the localization precision: A theoretical approach, that uses pre-known setup configurations in combination with information on the

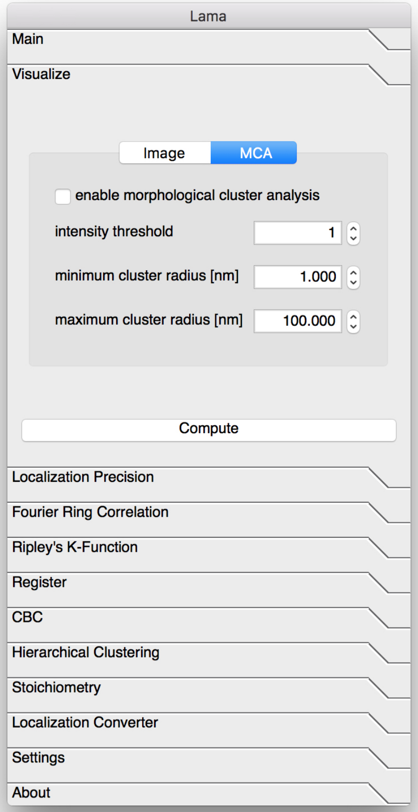

Figure 5. Lama MCA menu (Visualize cabinet)
Morphological Cluster Analysis (MCA) identifies cohesive regions within an image after threshold dependent background correction.

integrated intensity in order to calculate the localization precision obtained either by *least-squares estimation* (LSE) or *maximum likelihood estimation* (MLE), and a nearest neighbor based approach, that analyzes the localization distribution of the fluorescent probes being localized multiple times.

7.1 The Theoretical menu (Precision cabinet)

The theoretical localization precision is calculated from the integrated intensity of single PSFs using the methods of Thompson et al. [2002] and Mortensen et al. [2010]. It is therefore only valid if the fluorophore's PSF parameters were estimated by an LSE routine. The theoretically achievable precision of such a fitting procedure is described by equation:

$$\Delta = \sqrt{\frac{\sigma^2 + a^2/12}{N} + \frac{8\pi\sigma^4 b^2}{a^2 N^2}} \quad (2)$$

The localization precision depends mainly on the number of detected photons (N) and the standard deviation of the PSF (σ), but also on the area imaged in one pixel of the *Electron Multiplying Charged Coupled Device* (EMCCD) chip (a), and the background signal (b). Both σ and N depend on the optical setup characteristics as well as on the fluorescent label, while a depends on the configuration of the optical setup. As a single SMLM datas set contains usually only information of a single type of fluorescent probes at once, the the standard deviation of the fluorescent probe's PSF is fixed by the user as *mean sigma from psf*. The parameter for the background noise is fixed as *noise*. As equation (2) underestimates the realistic localization precision systematically by about 30%, it was later optimized by equation (3) by Mortensen et al. [2010].

$$\Delta = \sqrt{\frac{\sigma^2 + \frac{a^2}{12}}{N}\left(\frac{16}{9} + \frac{8\pi b^2(\sigma^2 + a^2/12)}{Na^2}\right)} \quad (3)$$

These methods assume an ideal microscopic setup without further aberrations except for a certain noise and give a rather optimistic value for the achieved localization precision. The realization of the theoretical estimation within Lama is shown in algorithm 4 (see also figure 6)

Both of these methods calculate the achieved localization precision for every detected localization and therefore fulfill the spirit of single molecule experiments, by enabling the detection of different subspecies within the ensemble of measured data.

In some cases a MLE of the molecule's PSF-parameters give superior a result compared to the parameters obtained by LSE fitting [Myung, 2003, Stallinga and Rieger, 2010].

Algorithm 4: Lama theoretical localization precision analysis algorithm

```
Data: single molecule localization file list L_ROI;
Input: standard deviation of the fluorophore PSF σ;
Input: setup pixel size a;
Input: background signal b;
Result: list of each localizations precision L_LocAcc;
Result: distribution of Thompson localization precision
        values D_Tho;
Result: distribution of Mortensen localization precision
        values D_Mor;
receive L_ROI from Algorithm 1;
create empty list of localization precision values
L_LocAcc;
for all single molecule localizations i in L_ROI do
    calculate localization precision after Thompson (eq:
    Ref) Acc_Tho(i);
    write write localization precision Acc_Tho(i) to
    L_LocAcc(i,0);
    calculate localization precision after Mortensen (eq:
    Ref) Acc_Mor(i);
    write write localization precision Acc_Mor(i) to
    L_LocAcc(i,1);
calculate mean value mean_Tho of L_LocAcc(:,0);
calculate mean value mean_Mor of L_LocAcc(:,1);
save mean_Tho, mean_Mor, and L_LocAcc;
create 1d histogram Hist_Tho of L_LocAcc(:,0);
save Hist_Tho;
create 1d histogram Hist_Mor of L_LocAcc(:,1);
save Hist_Mor;
```

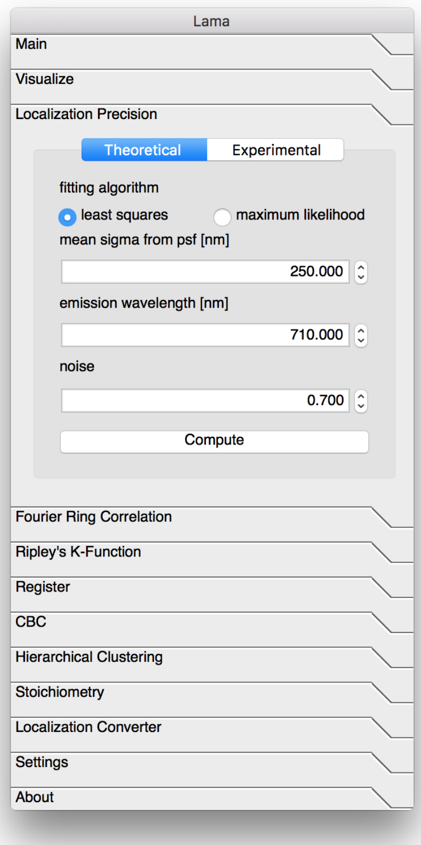

Figure 6. Lama Theoretical menu (Precision cabinet)
Calculates the localization precision of each localization by the method of Thompson [Thompson et al., 2002] and Mortensen [Mortensen et al., 2010].

This is particularly the case for SMLM experiments with a low background noise [Rieger and Stallinga, 2014]. Therefore, several localization fitting routines like rapidSTORM or ThunderStorm provide a MLE-based parameter estimation algorithm. In order to analyze localization data sets that are extracted from SMLM-experiments via MLE just switch the radio-button on top of the cabinet to *maximum likelihood* and Lama will calculate each fluorescent probes' localization precision after Rieger and Stallinga [2014].

$$\tau = \frac{2\pi b^2 \left(\sigma^2 + \frac{a^2}{12}\right)}{Na^2} \tag{4}$$

$$\Delta = \frac{\sigma^2 + \frac{a^2}{12}}{N} \left(1 + 4\tau + \sqrt{\frac{2\tau}{1+4\tau}}\right) \tag{5}$$

Notice, that in the original paper by Rieger and Stallinga [2014] b is defined as the number of background photons per pixel, while Lama defines b like Mortensen et al. [2010] as the noise of the background. That is why formula (5) differs from the original paper. Assuming shot noise statistics, the noise is the square root of the number of photons and therefore it needs to be b^2.

7.2 The Experimental menu (Precision cabinet)

In order to account for aberrations resulting from the microscopic setup, an experimental way of determining the localization precision was developed by Endesfelder et al. [2014]. Examining separated single molecules on a single molecule surface allow the determination of the localization precision by simply determining the center of mass from multiple localizations of the same fluorphore (see figure 7 a and b). The method presented here is based on analyzing the distribution of localizations originated from multiple fluorophores around their center of mass by calculating pairwise distances d_{ij} between the localizations resulting from the same fluorophore (see figure 7 c)

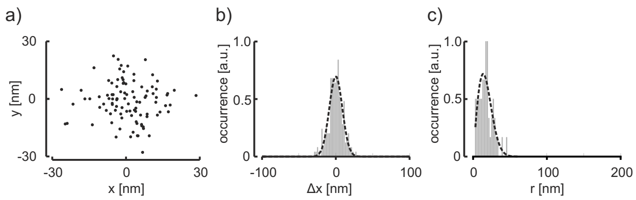

Figure 7. Single-molecule localization precision
a) Simulation of a single fluorescent molecule that is localized 100 times. b) The distance distribution from all localizations to the origin is described by a Gaussian function with $\sigma = 10.0(7)$ nm. c) The pairwise distance distribution between all localizations is described by equation (6) with $\sigma = 10.1(5)$ nm (input parameter: 100 simulations with 100 Gaussian distributed localizations with $\sigma = 10$ nm, graph was adopted from [Endesfelder et al., 2014])

The derivation of how to actually determine the localization precision of a Gaussian distribution from multiple point-to-point distances is described in section B.1.

During an SMLM experiment less localizations per molecule, as shown in figure 7, will be detected but several fluorophores will be observed. In order to average the localization precision over many fluorophores the following assumptions are made:

- The photo-physics of multiple fluorophores from the same population (e.g. Alexa Fluor 647) are expected to be equal.
- Only one population of fluorophores is analyzed; the probability density distribution $p_1(d)$ and $p_2(d)$ from two different fluorophores are expected to be equal $p_1(d) = p_2(d)$
- The on-time of the fluorophores is set to be in the range of the camera integration time.
- The blinking kinetics of the fluorophores are not in phase with the acquisition frame rate and most single molecule on-events are detected in two adjacent frames..

By assuming that the nearest neighbor of a specific localization within the adjacent frame belongs to the very same fluorophore and by approximating the localization distribution from every manifold of detected fluorophores being identical, it is possible to calculate the pairwise distance distribution $p(d_{ij})$ of an ensemble averaged over all fluorophores.

The technique is therefore termed *Nearest Neighbor in Adjacent frames* (NeNA) [Endesfelder et al., 2014]. As it only depends on distances, it does not require any further experimental parameters (see figure 8).

$$p(d_{ij}) = \left(\frac{d_{ij}}{2\sigma^2} e^{\frac{-(d_{ij}^2)}{4\sigma^2}} \right) \tag{6}$$

Here $p(d_{ij})$ describes the probability for a positional displacement d_{ij} in two dimensions and σ represents the standard deviation of the Gaussian distributed localizations.

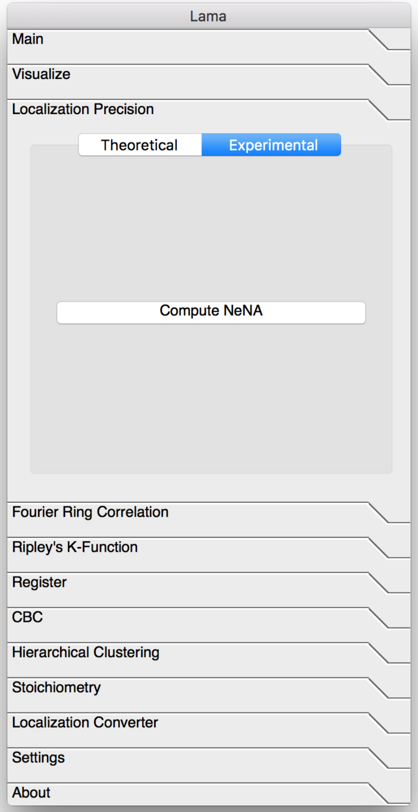

Figure 8. Lama Experimental menu (Precision cabinet)
NeNA calculates the mean localization precision over a localization ensemble of a SMLM experiment by measuring point to point distances of multiple localized fluorophores.

A certain fraction of nearest neighbor distances from adjacent frames stem from different molecules and will distort the distribution by contributing distances that are far beyond the expected localization precision. For an accurate fitting of the relative distances between localizations arising from the same molecule, two correction terms were conceived from Monte Carlo simulations: a first for short range distortions occurring from molecules within the direct neighborhood ($corr_{short}$), and a second for long range distortions ($corr_{long}$), which includes background noise.

$$P(d_{ij}) = p(singlemolecule) + corr_{short} + corr_{long} \tag{7}$$

$$\begin{aligned} P(d_{ij}) = {} & A_1 \left(\frac{d_{ij}}{2\sigma^2} e^{\frac{-(d_{ij}^2)}{4\sigma^2}} \right) \\ & + A_2 \frac{1}{\sqrt{2\pi\omega^2}} e^{\frac{-(d_{ij}-d_c)^2}{2\omega^2}} \\ & + A_3 d_{ij} \end{aligned} \tag{8}$$

The variables A_1, A_2, and A_3 represent free fitting parameters that describe the amplitude of each term; ω corresponds to the width of $corr_{short}$.

Algorithm 5: Lama experimental localization precision analysis algorithm

Data: single molecule localization file list L_{ROI};
Result: list of each localizations nearest neighbor in adjacent frame L_{NN};
Result: distribution of nearest neighbor in adjacent frame distances and NeNA fit D_{NeNA};
Result: mean localization precision σ_{NeNA};

```
receive L_ROI from Algorithm 1;
create empty list of nearest neighbor distances L_NN;
for all single molecule localizations i in L_ROI do
    extract frame number F(i) = t;
    determine distance d to nearest neighbor of all
    localizations in F(:) = t + 1;
    write d to L_NN(i,0);
create 1d histogram D_NeNA of L_NN;
determine σ_NeNA by fitting NeNA function (eq: Ref) to
D_NeNA;
save D_NeNA;
save L_NN and σ_NeNA;
```

NeNA reports the average localization precision of an SMLM experiment and is therefore limited to the analysis of a single fluorophore species. For multiple fluorophore species NeNA results in a superposition of individual distance distributions. This restricts the detection of subpopulations by localization precision.

On the other hand, the experimental NeNA approach offers three key advantages over the theoretical approaches: (i) it is based on the microscopic setup performance, including all aberrations as caused by imperfect optics; (ii) it is designed to calculate also the localization precision in axial dimension; (iii) it can be used to define the quality of drift correction and image registration, as these techniques add an additional localization precision error to the data set.

8. The Fourier Ring Correlation cabinet

The *Fourier ring correlation* (FRC) is a widely used method to determine the resolution of cryo-em images [Saxton and Baumeister, 1982]. The method was first established to analyze the actual resolution of an image reconstructed from an SMLM data by Nieuwenhuizen et al. [2013]. The data set is split into two data sets, equal in the number of localizations. Both data sets are used to reconstruct a super resolved image, which are then transferred into Fourier space. Starting from the center, the normalized cross-correlation coefficient between the segment of a circle from both Fourier images is calculated.

$$FRC(\omega) = \frac{\sum_{\omega_i \in [\omega, \omega+\Delta]} \hat{f}_1(\omega_i) \hat{f}_2(\omega_i)^*}{\sqrt{\sum_{\omega_i \in [\omega, \omega+\Delta]} |\hat{f}_1(\omega_i)|^2 \sum_{\omega_i \in [\omega, \omega+\Delta]} |\hat{f}_2(\omega_i)|^2}} \tag{9}$$

With ω being the spatial frequency in Fourier space and $\hat{f}_x$ representing the pixel in Fourier space. The * indicates for the complex conjugated of the Fourier transform. The procedure is repeated for circle segments with growing radius and constant segment width Δ. The resulting cross-correlation coefficient is plotted against the radial Fourier frequency of the inner segment edge. The resolution of an SMLM image is defined to be the smallest Fourier frequency at which the FRC drops beneath a value of $\frac{1}{7}$ [Nieuwenhuizen et al., 2013]. It can be calculated from the inverse Fourier frequency at the

intersection.

The Lama realizes the splitting of the SMLM data set by:

- *splitting into consecutive halfs*
- *sorting by odd and even indices*
- *splitting completely random*

The *FRC threshold* is pre-set to the value of $\frac{1}{7}$ but can be changed by the user.

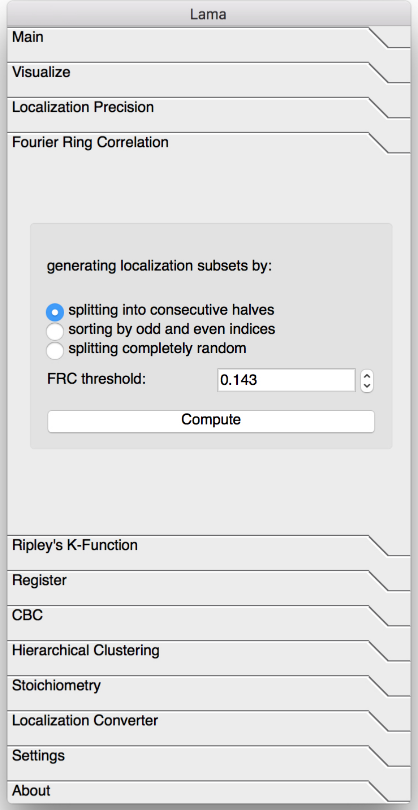

Figure 9. Lama FRC menu
Fourier ring correlation.

9. The Ripley's K-Function cabinet

The *Ripley's K-Function* cabinet (see figure 10) calculates a second order statistic over the variance of all inter-molecular distances r within a square ROI up to a maximal *radius of observation* [Ripley, 1976].

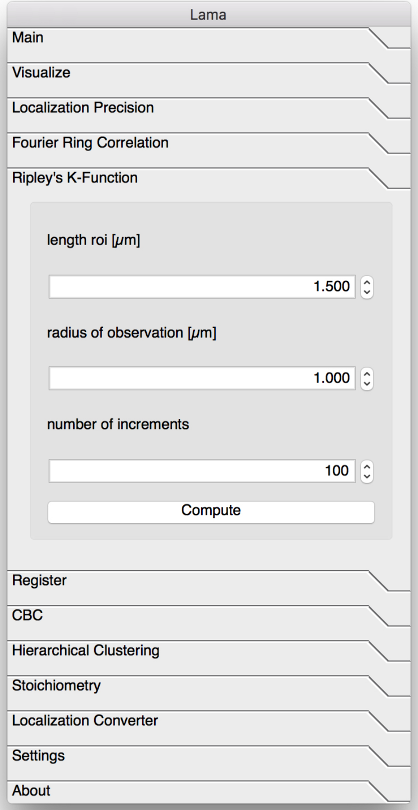

Figure 10. Lama Ripley's K-function cabinet
Analyze localization distribution against the hypothesis of being normally distributed by calculating Ripley's K-function. Choose the length of a ROI, defining the pool of localizations to analyze. Choose the observation radius for Ripley's K-function. Choose the number of distance increments for the calculation.

It tests a two-dimensional localization pattern against the null hypothesis of a uniform distribution and characterizes the degree of clustering [Ripley, 1976]. $K(r)$ calculates to:

$$K(r) = \frac{1}{n^2} \sum_i \sum_j \frac{N_r(d_{ij})}{\lambda} \tag{10}$$

With r being the radius, n being the total number of localizations, i being the walk parameter over the localizations, j being the walk parameter over the possible neighbors, λ being

a weighting factor for the area of investigated i, and $N_r(d_{ij})$ being the number of neighbor localizations within the distance r summed over all i and j. For the sake of a more intuitive interpretation, the K-function is linearized to the L-function:

$$L(r) = \sqrt{\frac{K(r)}{\pi}} \tag{11}$$

A further normalization to the H-function calculates to an expected value for all investigated distances r of the uniform distribution to:

$$H_{un}(r) = L_{un}(r) - r = 0 \tag{12}$$

In the case of a non-uniform distribution, $H(r)$ will exhibit a maximum. The distance $r_{\max}$ at which $H(r)$ has its maximum defines the average degree of clustering within the ROI (see figure 11 a). Typically $r_{\max}$ lies between the average cluster radius and the average cluster diameter, depending on the mean distance between the clusters (see figure 11).

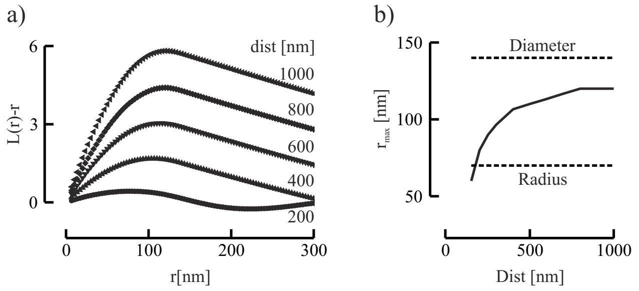

Figure 11. The degree of clustering
a) $H(r)$ was calculated for simulated clusters at different inter-cluster distances, ranging from 200 nm to 1 µm, leading to a shift in the maximum with increased inter-cluster distance. b) $r_{\max}$ is plotted against the inter-cluster distance. The graph shows that $H(r)$ yields a measure for the cluster size which depends also on the inter-cluster distance, and which generally scales between the cluster radius and the cluster diameter ($r = 75$ nm, graph adopted from Malkusch et al. [2013]).

A weakness of the second order statistic function is the appearance of edge effects. They occur if the distance of the investigated neighborhood exceeds the borders of the ROI; a part of the neighborhood localizations would be located outside the ROI and would therefore not exist in the pool of available neighboring candidates j. As a consequence, the number of detected neighbors would be underestimated. To correct for this, a qualified method of edge correction needs to be defined if using Ripley's K-function for cluster analysis.

9.1 Edge Correction in Lama

Lama uses toroidal edge correction (see section B.2) to test the degree of clustering against the null hypothesis of a uniform distribution.

Algorithm 6: Lama Ripley's K-function analysis algorithm

Data: localization file L;
Input: starting coordinates of ROI P;
Input: length of ROI len;
Input: maximal radius of observation $R_{\max}$;
Input: number of observation increments Inc;
Result: list of localizations within ROI L_{ROI};
Result: Ripley's K-, L-, and H-function L_{Rip};
Result: the radius of Ripley's H-function's amplitude r_{max};

create empty list of localizations L_{ROI};
load single molecule localization list L;
from P and len create sorting parameter to create square ROI S_{ROI} **for** *all single molecule localizations I in L* **do**
    compare I with S_{ROI};
    if *I fits S_{ROI}* **then**
        write I to L_{ROI};
save L_{ROI};
create empty list of Ripley's K-function L_{Rip};
for *all single molecule localizations I in L_{ROI}* **do**
    write all neighbors within distance $R_{\max}$ to J;
    for *all distances r[0:Inc:R_{max}]* **do**
        detect number of neighbors $N_r(d_{IJ})$;
calculate $K(r)$ by weighting $N_r(d_{IJ})$ for the total number of localizations n and the area λ;
linearize $K(r)$ to $L(r)$;
normalize $L(r)$ to an all distance expectation value of 0 for a uniform distribution to $H(r)$;
save $K(r)$, $L(r)$ and $H(r)$;
plot $K(r)$, $L(r)$ and $H(r)$;

Ripley's K-function is usually used to characterize the

distribution of proteins [Owen et al., 2010, Lillemeier et al., 2010, Malkusch et al., 2013, Muranyi et al., 2013, Truan et al., 2013]. It was also used to characterize photo-physics, as multiple localizations of a photo-switching fluorophore result in small micro-cluster [Endesfelder et al., 2011].

10. The Register cabinet

All SMLM experiments suffer from several types of image aberrations. When imaging only a single channel these aberrations are usually neglected, due to the lack of a reference structure. When it comes to multi-color SMLM experiments (wether recorded with a parallel or sequentially) the localization files of the spectral channels often show various facets of image aberrations. The sources of these image aberrations are versatile, and it is not recommended to calculate a setup-specific correction function. Usually, a registration of two channels is performed based on the distribution of multicolor fiducial markers. The markers are detected in both channels, paired, and from the paired positions a transformation matrix is calculated. The quality of registration follows a simple rule: The more fiducial marker positions are paired, the better the registration will work. As image aberrations will not follow linearities the best results will be achieved by non-linear transformation matrices. However, these matrices are based on a large number of fiducial marker position pairs. The calculation of such a matrix can be realized by preparing a calibration sample consisting of a surface with multi-spectral beads [Churchman et al., 2005, Malkusch et al., 2012]. This requires to change samples in between measuring the calibration sample and the actual sample, which may cause slight changes due to minimal setup instability resulting in additional aberrations. A second approach is the use of fewer fiducial markers that are immobilized on the sample directly. From these markers an affine transformation matrix is calculated correcting for image aberrations and channel translation. It is our experience that implementation of the latter method is robust in SMLM experiments [Zessin et al., 2013].

Lama splits the registration routine into two approaches: The fiducial marker detection approach, that results in a localization list of fiducial marker positions, and the registration approach; here a localization file is registered based on a translation matrix that is calculated from two fiducial marker localization lists.

10.1 The Bead Detection menu (Register cabinet)

Lama detects fiducial markers by an MCA analysis. Usually a fiducial marker is characterized by a high photon yield and is detected in almost every frame. Multiple detections of the same marker will therefore form small and dense *focal adhesion site* (FAS). Possible candidates are extracted from a MCA file by their minimal and maximal *cluster radius* and the number of frames, the marker is detected in (*on-time*, see figure 12).

10.2 The Registration menu (Register cabinet)

The centroid position of FASs in both channels are linked into coordinate pairs $\vec{p}$ and $\vec{p}'$. In order to prevent a false pairing a *maximal displacement* distance is chosen (see figure 13). From these coordinate paris an affine transformation matrix $\hat{M}$ is calculated. In order to calculate an appropriate affine correction matrix the data must at least contain three fiducial markers identified in both channels.

Algorithm 7: Lama Focal Adhesion Site detection algorithm

Data: localization file L_{ROI};
Input: minimal radius of FAS r_{min};
Input: maximal radius of FAS r_{max};
Input: radius of observation r_{obs};
Input: minimal number of localizations I_{min};
Result: list of vectors containing FAS centroid positions L_{FAS};

receive L_{ROI} from Algorithm 1;
create empty list of FAS centroid positions L_{FAS};
create empty list cohesive regions L_{cluster};
create 2D-hitogram I_{raw} of L_{ROI};
save I_{raw};
create binary image I_{bi} by applying $thr = 1$ to I_{raw};
detect list of cohesive regions L_{cluster} from I_{bi};
for *all i in* $L_{cluster}$ **do**
    if $r_{min} \leq r(i) \leq r_{max}$ **then**
        transpose cohesive regions to I_{raw};
        extract centroid coordinates $\vec{p}(i)$;
        detect number of neighbors I in distance r_{obs} around $\vec{p}(i)$;
        if $I \geq I_{min}$ **then**
            write $\vec{p}(i)$ to L_{FAS}
save L_{FAS}

$$\hat{M}\vec{p} = \vec{p}' \tag{13}$$

$$\begin{pmatrix} a & b & dx \\ c & d & dy \\ 0 & 0 & 1 \end{pmatrix} \begin{pmatrix} x \\ y \\ 1 \end{pmatrix} = \begin{pmatrix} x' \\ y' \\ 1 \end{pmatrix} \tag{14}$$

Here $\left(\begin{smallmatrix} a & b \\ c & d \end{smallmatrix}\right)$ represents the part of $\hat{M}$ that corrects for image aberrations and dx and dy correct for the translation between none perfectly aligned imaging planes.

Image registration by no means is a correction for aberrations in an absolute way (which would mean guiding you one step closer to the ground truth of the emitter distribution at your target structure). It rather adjusts the image aberrations of one channel to the image aberrations of the other.

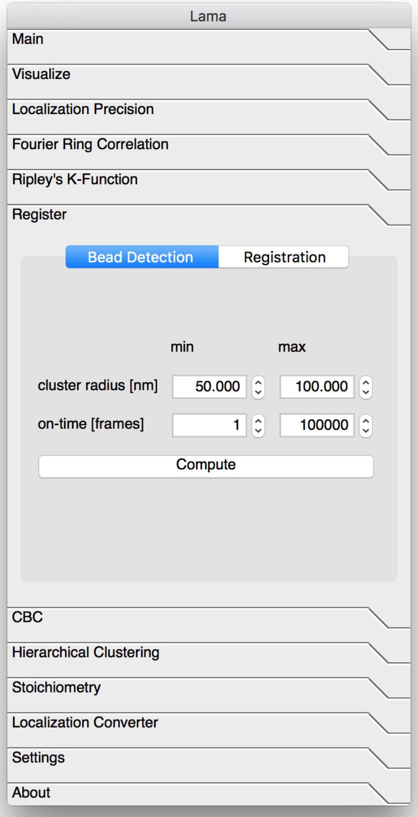

Figure 12. Lama Bead Detection menu (Register cabinet) detect the position of fiducial markers in an SMLM experiment. Choose the minimal FAS radius. Choose maximal FAS radius. Choose the radius of observation, within localizations are detected around a FAS. Choose the relative on-time of the fiducial markers to determine the expected number of localizations per marker. Choose the expected displacement between the two channels.

11. The CBC cabinet

Colocalization is usually used as an equivalent for the probability of two species of proteins to undergo an interaction with each other. The Journal of Histochemistry and Cell Biology claims in it's annual review of 2012 that "*the introduction of sub-diffraction-limited, super-resolution microscopy techniques has engendered a paradigm shift in approaching the colocalization analysis of single molecules*" [Taatjes and Roth, 2013]. This means that neither of the conventional methods of measuring colocalization suites for the data obtained from a

Algorithm 8: Lama registration algorithm

Data: list of localizations channel 2 L_{cha2};
Data: list of FAS centroids channel 1 L_{FAS1};
Data: list of FAS centroids channel 2 L_{FAS2};
Input: distance threshold of translation between both channels D_{max};
Result: list of registered localizations for channel 2 L'_{ch2};

create empty transformation matrix $\hat{M}$;
create empty list of registered localizations for channel 2 L'_{ch2};
link L_{FAS1} to L_{FAS2} by nearest neighbor analysis in respective channel;
delete FAS centroids without partner;
create linear system of equations $\hat{M}L_{\text{FAS1}} = L_{\text{FAS2}}$;
estimate $\hat{M}$ by solving linear system of equations;
transform $\hat{M}L_{\text{ch2}} = L'_{\text{ch2}}$;
save L'_{ch2};

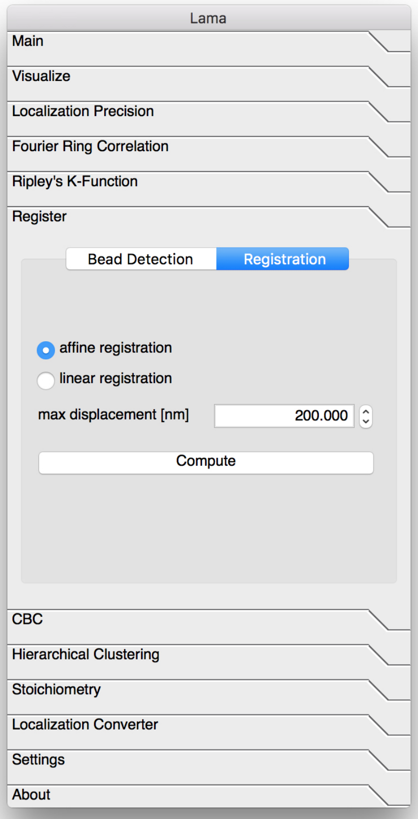

Figure 13. Lama Registration menu (Register cabinet)
Register two channels on top of each other by a distribution of fiducial markers. Choose the method of registration. For an affine registration at least three fiducial marker positions are needed.

localization-based super-resolution fluorescence microscopy experiment. Conventional measurements of colocalization would analyze overlapping PSFs in diffraction-limited fluorescence images of spectrally separate channels as an overall ensemble measurement. *Foerster Resonance Energy Transfer* (FRET) experiments allow to obtain single molecule data of protein-protein interaction for a molecular separation below 10 nm. In order to close the gap between diffraction-limited fluorescence imaging and single molecule FRET measurements, we developed a coordinate-based approach to indicate colocalization at a single molecule level [Malkusch et al., 2012]. The *coordinate based colocalization* (CBC) approach defines colocalization of two molecular fractions by a similar molecule distribution at the microscopic level.

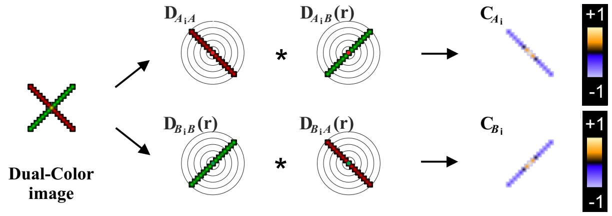

Figure 14. The CBC concept
CBC for a simulated distribution of single-molecule localizations: For each single-molecule localization of either species (green or red), the distribution function of the neighboring localizations from the same species is calculated. In a second step, the distribution function of the neighboring localizations from the other population is calculated. From the individual distribution functions, a correlation coefficient is calculated and weighted by the distance distribution. As a result, each single- molecule localization of each species is attributed an individual colocalization value, which reports on the local environment of a particular red or green molecule, separately (Graph adopted from Malkusch et al. [2012]).

The CBC routine calculates a value for each localization A_i out of two spectrally separated single molecule localization lists (A and B) by calculating the neighborhood distributions of both channels ($D_{A_i,A}$ and $D_{A_i,B}$) around A_i, ranging from

$r_0 = A_i$ to $R_{\max}$ (see figure 14, left).

$$D_{A_i,A} = \frac{N_{A_i,A}(r)}{\pi r^2} \cdot \frac{\pi R_{\max}^2}{N_{A_i,A}(R_{\max})} = \frac{N_{A_i,A}(r)}{N_{A_i,A}(R_{\max})} \cdot \frac{R_{\max}^2}{r^2} \tag{15}$$

$$D_{A_i,B} = \frac{N_{A_i,B}(r)}{N_{A_i,B}(R_{\max})} \cdot \frac{R_{\max}^2}{r^2} \tag{16}$$

With $N_{A_i,A}(r)$ being the number of neighbors from fraction A at distance r around A_i, and $N_{A_i,B}(r)$ being the number of neighbors from fraction B at distance r around A_i. πr^2 is a linearization factor. $N_{A_i,A}(R_{\max})$ and $N_{A_i,B}(R_{\max})$ are normalization factors that account for the maximal number of neighbors within the distribution. In order to compare the distribution of the two protein fractions Lama calculates the rank correlation coefficient after Spearman to $S_{\mathrm{A,B}}(i)$ (see figure 14, right).

$$S_{A_i} = \frac{\sum_{r_j=0}^{R_{\max}} \left(O_{D_{A_i,A}}(r_j) - \overline{O}_{D_{A_i,A}}\right)\left(O_{D_{A_i,B}}(r_j) - \overline{O}_{D_{A_i,B}}\right)}{\sqrt{\sum_{r_j=0}^{R_{\max}} \left(O_{D_{A_i,A}}(r_j) - \overline{O}_{D_{A_i,A}}\right)^2}\sqrt{\sum_{r_j=0}^{R_{\max}} \left(O_{D_{A_i,B}}(r_j) - \overline{O}_{D_{A_i,B}}\right)^2}} \tag{17}$$

With $O_{D_{A_i,A}}(r)$ being the rank of $D_{A_i,A}(r)$ with an arithmetic average of $\overline{O}_{D_{A_i,A}}$. With $\overline{E}_{A_i,B}$ being the distance between A_i to its nearest neighbor from channel B the CBC value C_{A_i} for a single localization A_i calculates to:

$$C_{A_i} = S_{\mathrm{A,B}}(i) \cdot e^{\frac{\overline{E}_{A_i,B}}{R_{\max}}} \tag{18}$$

The radius of observation $R_{\max}$ should be typically chosen to a value below the diffraction limit. Increasing the number would lead to a more precise measurement but also increases the calculation time. changing the weighting factor $\overline{E}_{A_i,B}$, one can define if the CBC analysis should be more weighted on the distance factor or on the neighborhood correlation factor. These factors have to be chosen for every type of data set and need to be identical for the sake of comparability of different measurements.

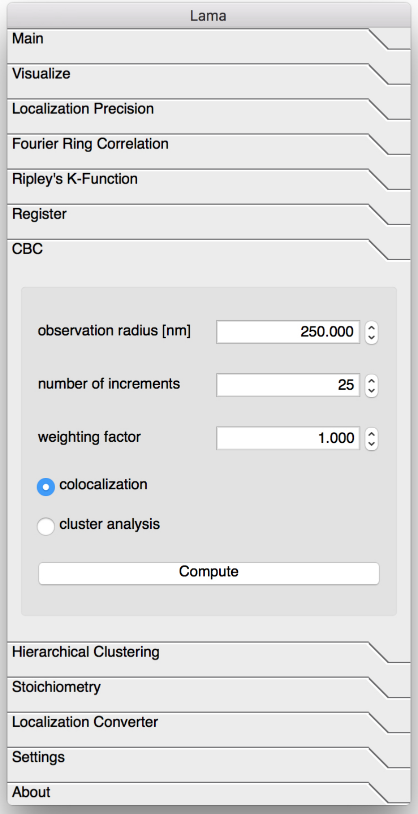

Figure 15. Lama CBC cabinet
Calculate a coordinate based coloalization for two species of single molecule SMLM data. The observation radius indicates the distance around every localization for the CBC calculation. The weighting factor weighs the CBC value for the distance to the nearest neighbor in the opposite species. By choosing *cluster analysis* a single SMLM data set is split in half by separating localizations occurring in frames with odd numbers from those occurring in frames with even numbers. These two species are CBC analyzed afterwards.

A CBC analysis attributes a CBC value to each localization. It returns a localization list for both of the two channels. The CBC value indicates the possibility that this very localization does ($1 \geq C_{A_i} > 0$) or does not colocalize ($0 \geq C_{A_i} \geq -1$) with a localization from the other channel.

The CBC result files may be reloaded into Lama. By choosing the *Use CBC File* checkbox the computed images will show a two-dimensional colocalization distribution map. Furthermore, localization lists containing CBC information can be pre-sorted to visualize only the colocalizing fraction.

11.1 CBC mediated cluster analysis

CBC can also be used for cluster analysis. By splitting the localization list in even and odd frame numbers the CBC algorithm can be used to correlate every localizations' neighborhood with itself. This procedure reports whether a protein is uniformly distributed at the microscopic scale, which is usually the case in dense clustered regions. The CBC value indicates the degree of clustering for every localization's environment. As the normal CBC value, this index can be used to sort every localization into those arranged within a cluster, or those that are not. This way the CBC algorithm offers also the possibility for a coordinate-based cluster analysis that, compared to Ripley's K-function [Ripley, 1976] or the pair correlation *photo activated localization microscopy* (PALM) algorithm [Sengupta et al., 2011, Sengupta and Lippincott-Schwartz, 2012], also contains spatial information.

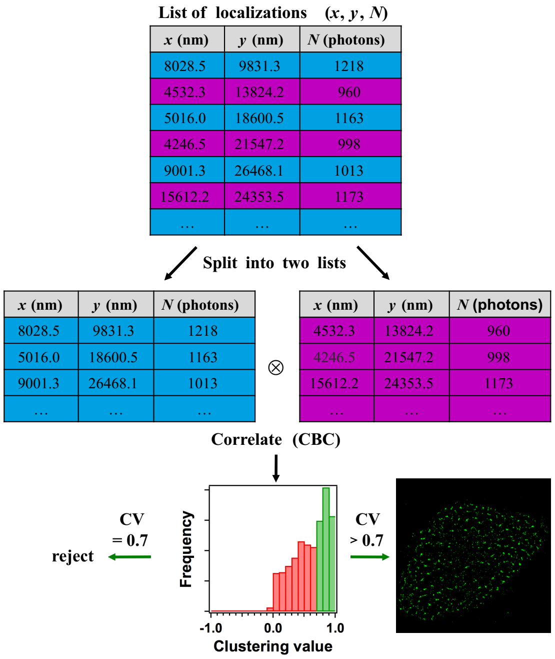

Figure 16. CBC mediated cluster analysis
The localization list is split into two lists comprising only localizations with even, or odd frame numbers. The resulting localization lists are correlated by the CBC algorithm in order to determine each localizations degree of clustering. By applying a threshold, a binary clustering map is calculated form all localizations characterized by a $C_{A_i} > 0.7$ (Graph adopted from Díez et al. [2014]).

12. The Hierarchical Clustering cabinet

Hierarchical clustering is a method that sorts localizations into clusters based on local density. Cluster expansion might occur if localizations are placed close to cluster regions. By expanding the microenvironment, hierarchical clustering is able to connect cluster regions of all kind of shape (see figure 17).

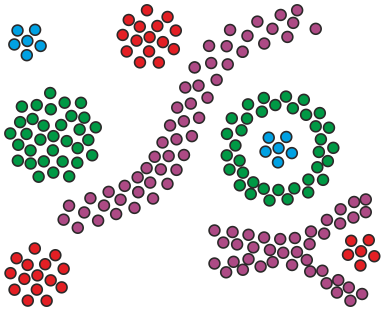

Figure 17. Hierarchical clustering
By expanding the microenvironment, hierarchical clustering is able to connect cluster regions of all kind of shape, despite from gaussian.

12.1 The Sort menu (Hierarchical Clustering cabinet)

Hierarchical clustering is implemented in Lama by the *Density-Based Algorithm for Discovering Clusters in Large Spatial Databases with Noise* (DBSCAN). The theory of this algorithm can be found in the work of Ester et al. [1996]. Briefly, DBSCAN sorts every localization from the localization list into one of three categories: core cluster localization, edge localization, noise localization (see figure 19). A core cluster localization is defined as a localization with at least $P_{\min}$

neighbors within an observation radius of ε (see figure 18 and 19, red points).

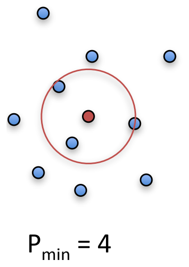

Figure 18. The definition of a core localization
A core localization is defined as a localization, that has at least $P_{\min}$ neighbors within the observation radius ε. The example shows ε as a red circle around a core localization with $P_{\min} = 4$.

Edge localizations are defined as localizations that have less than $P_{\min}$ neighbors within the distance of ε are no core localization, but at least one neighbor that is a core localization. It is therefore termed to be density-reachable by a core localization (see figure 19, yellow points). If a localization has less than $P_{\min}$ neighbors within the distance of ε and also is not density-reachable by a core localization, it is defined as a noise localization (see figure 19, black points).

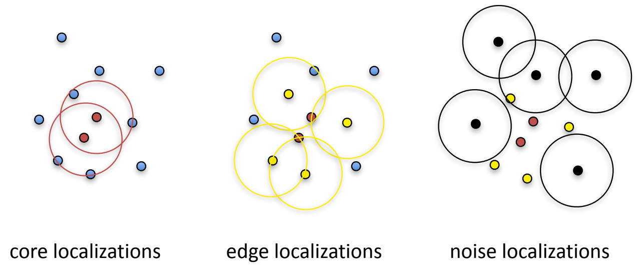

Figure 19. Sorting localizations into cluster types
By the density parameters $P_{\min}$ and ε all localizations are sorted into three types (core localizations, edge localizations, and noise localizations). Core localizations are defined as localizations that are surrounded by at least $P_{\min}$ neighbors within a radius ε. Edge localization are density-reachable from a core localization but have less than $P_{\min}$ neighbors within the distance ε, noise localizations have not enough neighbors to be a core localization and are not reachable from other core localizations.

The algorithm starts with a randomly-chosen localization and sorts it into one of the three categories (see algorithm 9). It continues until it finds the first core localization. From there it starts to expand the identified cluster. Expansion is done by sorting all the directly density-reachable neighbors of the core localization (see algorithm 10). If neighboring localizations are identified to be either a core localization or an edge localization, they are added to the cluster. If they are a core localization, their neighbors are added to the density-reachable neighbor list. This procedure is continued until there are no more localizations density-reachable from any localization within the cluster. The routine then searches for a new cluster until each localization was at least sorted once by the algorithm (see algorithm 9).

Algorithm 9: Lama DBSCAN analysis algorithm (adapted from *ref*)

Data: localization file L;
Input: minimal number of localizations to form a cluster $P_{\min}$;
Input: minimal reach distance ε;
Result: list of localizations sorted into clusters L_{ROI};

```
receive L_ROI from Algorithm 1;
set L_ROI.cID to unclassified;
ClusterID = 1;
for all i in L_ROI do
    if L_ROI(i).clusterID ==unclassified then
        if expand(L_ROI, L_ROI(i), ClusterID, P_min, ε)==true then
            ClusterID += 1;
save L_ROI;
```

In order to choose qualified sorting parameters ($P_{\min}$, ε) some knowledge about the experiment is needed. The density in an SMLM experiment depends on several factors that may have different origins. The first impact would be the protein distribution that should be characterized. Next, the labeling density depends mainly on the labelling strategy and is influenced by the labeling efficiency as well as by the length of the linker. A longer linker could result in a less dense distribution at the near-molecular scale. The degree of labelling of the linker

Algorithm 10: Lama Expand cluster analysis algorithm (adapted from *ref*)

Data: localization file L_{ROI};
Input: starting point $L_{\mathrm{ROI}}(i)$;
Input: ClusterID;
Input: minimal number of localizations to form a cluster $P_{\min}$;
Input: minimal reach distance ε;
Result: boolean if clustering is true C_{truth};

receive L_{ROI} from Algorithm 9;
seeds=L_{ROI}.regionQuery($L_{\mathrm{ROI}}(i)$,ε);
if *seeds.size* $< P_{min}$ **then**
    L_{ROI}.changeClusterID($L_{\mathrm{ROI}}(i)$,Noise);
    C_{truth}=FALSE;
else
    L_{ROI}.changeClusterIDs(seeds,ClusterID);
    seeds.delete($L_{\mathrm{ROI}}(i)$);
    while *seeds.size <> 0* **do**
        Point = seeds.first;
        result = L_{ROI}.regionQuery(Point, ε);
        if *result.size* $>= P_{min}$ **then**
            for *all j in result.size* **do**
                if *result(j).ClusterID == unclassified or noise* **then**
                    if *result(j).ClusterID = unclassified* **then**
                        seeds.append(result(j));
                    L_{ROI}.changeClusterID(result(j),ClusterID);
        seeds.delete(Point);
    C_{truth}=TRUE;
return C_{truth};

will also have an influence. Last but not least, the fluorophore that is used will also influence the density: a fluorophore with a high blinking probability will be detected multiple times during a single SMLM experiment, which would increase the density of localizations in the cluster. Furthermore there will also be an impact of the brightness from the fluorophore. As the number of photons collected from a single fluorophore during a switching cycle are directly connected to the localization precision (see equation (2) and (3)). This will directly influence the broadness of the the localization distribution.

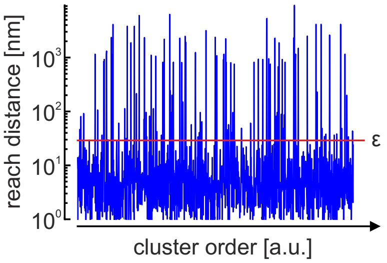

Figure 20. Ordered distribution of reach distances
Based on a given neighborhood parameter $P_{\min}$ the reach distance ε_i is calculated for each localization. The distribution is plot and a qualified observation radius ε is indicated by the red line.

Without all of this pre-knowledge it might be easier to guess the fraction of localizations that are most probably not organized in clusters by analyzing the SMLM image by eye. In order to enable the user for this image-based guess, we implemented the *Ordering Points To Identify the Clustering Structure* (OPTICS) algorithm to the Lama. The algorithm is described in detail by Ankerst et al. [1999]. Briefly, the user chooses just a minimal number of localizations $P_{\min}$. Then the OPTICS algorithm calculates for each localization i in your list the reach distance ε_i so that i would be a core localization (see figure 20).

Afterwards, a distribution of all reach distances is calculated and normalized to unity (see figure 21). From this probability distribution of the reach distance the user can now choose an observation radius ε for a DBSCAN analysis by thresholding

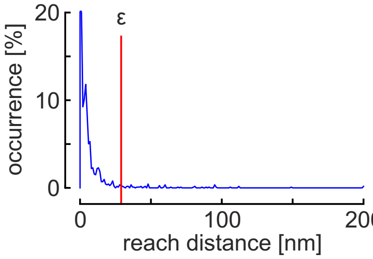

Figure 21. Distribution of all detected reach distances
From the distribution of all reach distances within a SMLM experiment a qualified guess for ε can be made, by indicating the fraction of localizations that are to be identified as core localizations. These localizations will have a shorter or equal reach distance than ε (here the fraction is 90 %).

the distributions into two subpopulations: A first fraction that would fulfill the criterion to be a core localization, and a second fraction that would not. In the given example we set the threshold so that a fraction of 0.9 would be a core localization indicating an expected high degree of clustering.

13. The MCA menu (Hierarchical Clustering cabinet)

From the result of a DBSCAN- or OPTICS-based hierarchical cluster analysis it is also possible to perform an MCA. For each defined cluster Lama comprises all localizations that were assigned to the cluster in order to calculate the cluster population. Furthermore, the edge localizations of the cluster are identified using a *Quickhull* algorithm [Barber et al., 1996], that detects the minimal polygon, which encloses all of the given points of acluster. From the edge localization the cluster area is calculated using the survey's area formula [Meister, 1769]. The results are saved in the same format as done by the image based MCA analysis. And can also be used for bead detection and registration.

Activating the *condense localization list before analysis* checkbox will search for localization assigned to the same area that are detected in consecutive frames. These localizations are most likely originated from the same fluprophore and are condensed to a single localization at the average position.

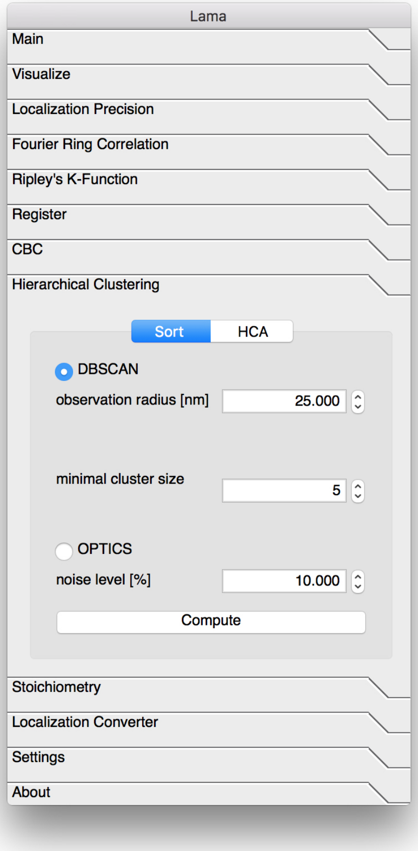

Figure 22. Lama Sorting menu (Hierarchical Clustering cabinet)
All localizations from a SMLM experiments are sorted individually into distinct clusters. Choose the observation radius ε, the minimal number of neighbors to define a core localization P_{min}. If using OPTICS, choose the fraction of localizations that will not be defined as a core localization in order to determine ε.

14. The stoichiometry cabinet

Probing the stoichiometry of a molecular complex is challenging, since individual molecules in an oligomer typically cannot be resolved spatially by SMLM. A possible solution to this problem is achieved based on the analysis of the intrinsic blinking of *photo-switchable* (ps)- *fluorescent protein* (FP)s within a diffraction-limited cluster of multiple localizations by a pre-known blinking model (The theory of molecular

counting is derived in section B.3).

14.1 Probing the oligomeric state of reversibly photo-switching fluorophores

In the case of multiple emitters N with each described by the same blinking parameter P_0, the number of detected blinking events n is described by the sum of the independent, geometric, random variables of single emitter blinking ($X_{D,i}$). This method was first introduced by Lee et al. [2012] and later used by Fricke et al. [2015] to dezermine membrane protein stoichiometries.

$$n = \sum_i X_{D,i} \tag{19}$$

The distribution of geometric, random variables, such as shown in equation (19), is described by the negative binomial distribution:

$$P_{N,P_0}(n) = \binom{N+n-1}{N-1} P_0^N (1-P_0)^n \tag{20}$$

Figure 23 shows the blinking probability distribution for various scenarios of multiple emitters ($N = 1,2,3$).

The generalized blinking distribution given by equation (20) turns into equation (92) for the special case of a single emitter ($N = 1$).

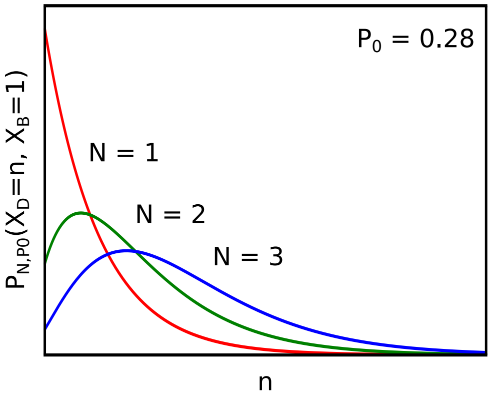

Figure 23. Blinking probability distribution of multiple emitter scenarios
The negative binomial distribution of the distribution of geometric, random variable $n = \sum_i X_{D,i}$ for multiple emitters N, all characterized by the same blinking parameter P_0, as described by equation (20)

14.2 Determining oligomeric states with Lama

Usually the number of blinking events $n = \sum_i X_{D,i}$ are determined from the number of individual detected emitter events within a diffraction limited area (see figure 24 top). Lama uses a cluster analysis file in MCA format as an input parameter and calculates the distribution of n from the intensity column that comprises the number of detected localization events within single cluster (see figure 24, bottom).

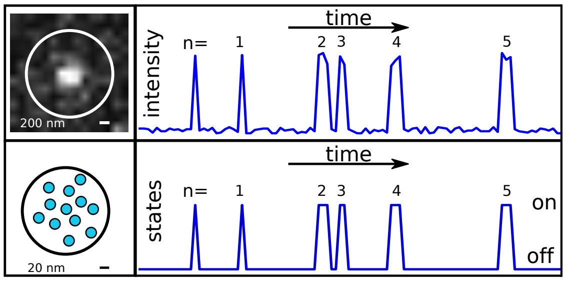

Figure 24. Detecting blinking events
(*top, left*): The detection of single molecule blinking events within a diffraction limited spot. (*top, right*): The intensity within the circular ROI is measured over time. The intensity time trace shows 5 blinking events. (*bottom left*): Hierarchical cluster analysis of all localizations form the top image reveals a cluster, comprising 12 localizations. (*bottom, right*): Binary state trace of all cluster localizations against time shows the number of blinking events.

In order to compensate for overcounting of molecules that are detected multiple times (because they are in the active state A during several adjacent frames without switching to the dark state D), the MCA should be performed with the *condense localization list before analysis* checkbox activated (see section 13). The calculated distribution of n is then fitted to equation (20). As the basis of the analysis is the probability P($X_D = n$, $X_B = 1$) for a single molecule to show $X_D = n$ blinking events,

and exactly one bleaching event $X_B = 1$, the experimental data has to be acquired until every detected molecule is photobleached.

A preliminary but unavoidable test, before determining oligomeric states, is the determination of the blinking parameter P_0, which is characteristic for each type of ps-FP. For this purpose a measurement of a protein complex with a known stoichiometry factor N is needed, the MCA analysis is performed, and the distribution is fitted to equation (20) with the known parameter N kept fix. For the single protein mEos2, a value of $P_0 = 0.28$ was determined [Fricke et al., 2015]

The user should keep in mind that the determination of oligomeric states as performed by Lama is based on the Markov chain model of blinking, as described in figure 29. This model is suitable for a large number of ps-FP but might not be suitable for all kinds of fluorophores.

15. The Localization Converter

In order to guarantee the free choice of software that generates localization data sets from raw image data, Lama provides a converter tool (see figure 25). By now, a number of *formats* can be ported directly, but we will provide more in the future. However, if the structure of an SMLM data set is known, a user defined *format* can be converted by choosing the *delimiter*, the sign that indicates for *comments*, and the *spatial unit* (like pixel of nano meter). Further, Lama needs to know which columns of the source file correspond to the x and y coordinate, the localization time (t) and the integrated intensity of the PSF (i).

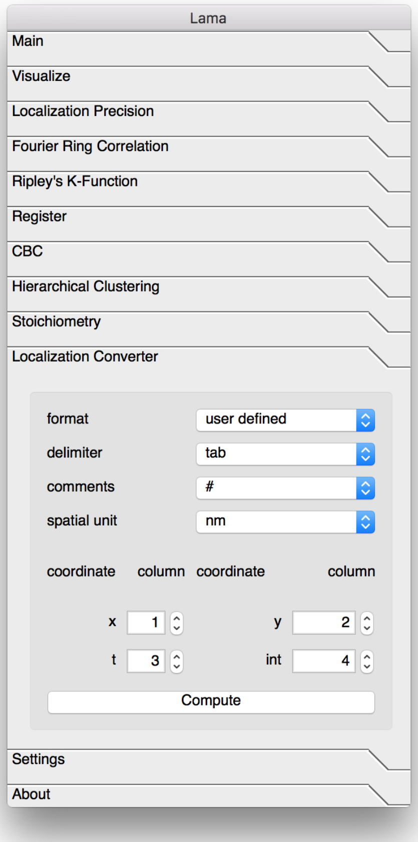

Figure 25. Lama Localization Converter menu
Convert SMLM data into MALK format.

16. The Settings cabinet

Analyzing SMLM data is very time-consuming because of a large number of localization in a localization list. In order to guarantee for a high reproducibility of these procedures Lama offers the possibility to store all used parameters for a distinct analysis session, such that no information gets lost. The procedure also allows to load the stored parameters in order to analyze the same data set by multiple different parameter sets and store the results into several folders.

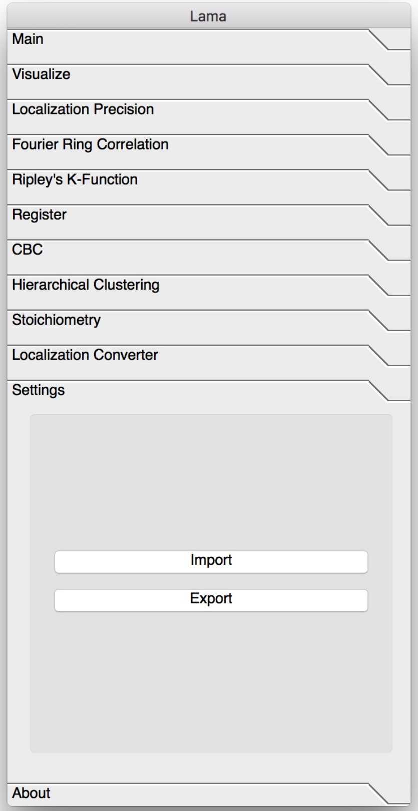

Figure 26. Lama Settings menu
Store or load the settings of the actual Lama session. Choose a folder to store the data.

Appendices

1. Outlook

The Lama project startet in late 2014 and is continuously evolving. In this section we present an overview about the current development process, that shall be provided with the next version of Lama.

A.1 Ubuntu Linux support

We are currently working on a stable version of a pre-compiled standalone deb-package for Ubuntu Linux, as well as an rpm-package for openSUSE.

A.2 Drift Correction

We developed a drift correction routine based on single particle tracking of a set of fiducial markers, that we are implementing at the moment [Zessin et al., 2013].

A.3 3D Data Sets

At the current state of development Lama analyzes 2D SMLM data sets. The reason for this is the wide choice of optical methods to obtain 3D spatial information from single fluorophores [Huang et al., 2008, Pavani et al., 2009, Juette et al., 2008, Shtengel et al., 2009]. All of these methods have in common that the lateral localization precision is distinct from the axial localization precision. This way a conservative extension of all coordinate-based cluster algorithms from a 2D plane into 3D space, without concerning the different localization precisions for each dimension, would be easy, but it would also lead to misinterpretation of the SMLM data. To further complicate the development of 3D post-processing routines, all of these methods additionally differ among each other in axial localization accuracy, making a simple solution only valid for one of them. Therefore, 3D post-processing of SMLM data with Lama is still in progress [Zessin et al., 2013] but will hopefully be supported soon.

A.4 Moving to c++

For reasons of performance we are planing to migrate the complete Lama project from an interpreted script language to a fully compiled program written in c++.

2. Theory

B.1 The theory of NeNA

The derivation of how to actually determine the localization precision of a Gaussian distribution form multiple point to point distances is described in the following lines starting from Fick's second law of diffusion.

$$\left(\frac{\delta}{\partial t}c\right)_{x,y} = D\left(\Delta c\right)_t \tag{21}$$

with Δ being the Laplace-Operator:

$$\nabla = \hat{e}_x\frac{\partial}{\partial x} + \hat{e}_y\frac{\partial}{\partial y} \tag{22}$$

$$\Delta = \nabla^2 \tag{23}$$

$$\Delta = \hat{e}_x\frac{\partial^2}{\partial x^2} + \hat{e}_y\frac{\partial^2}{\partial y^2} \tag{24}$$

For a single molecule, equation (21) calculates to:

$$\left(\frac{\delta}{\partial t}p\right)_{x,y} = D\left(\Delta p\right)_t \tag{25}$$

$$\tag{26}$$

With p representing the probability density of the localization. For further simplification we will define

$$p_t = \frac{\partial}{\partial t}p \tag{27}$$

$$p_{rr} = \frac{\partial^2}{\partial x^2}p + \frac{\partial^2}{\partial y^2}p \tag{28}$$

Equation (25) can be solved in Fourier space:

$$\begin{aligned}\hat{p}_t &= \mathscr{F}\{p_t(t,x,y)\}\\ &= \int_{-\infty}^{\infty}\int_{-\infty}^{\infty} e^{i(\omega x+\theta y)}p_t(t,x,y)\,dx\,dy\end{aligned} \tag{29}$$

$$\begin{aligned}D\hat{p}_{rr} &= \mathscr{F}\{Dp_{rr}(t,x,y)\}\\ &= \int_{-\infty}^{\infty}\int_{-\infty}^{\infty} De^{i(\omega x+\theta y)}p_{rr}(t,x,y)\,dx\,dy\end{aligned} \tag{30}$$

with:

$$\left(\frac{\partial^2}{\partial x^2} + \frac{\partial^2}{\partial y^2}\right)e^{i(\omega x+\theta y)} = (-\omega^2-\theta^2)e^{i(\omega x+\theta y)} \tag{31}$$

equation (30) caculates to:

$$\begin{aligned}D\hat{p}_{rr} &= -D\left(\omega^2+\theta^2\right)\\ &\int_{-\infty}^{\infty}\int_{-\infty}^{\infty} e^{i(\omega x+\theta y)}p(t,x,y)\,dx\,dy\\ &= -D\left(\omega^2+\theta^2\right)\hat{p}(t,\omega,\theta)\end{aligned} \tag{32}$$

So we end up with:

$$\hat{p}_t(t,\omega,\theta) = -D\left(\omega^2+\theta^2\right)\hat{p}(t,\omega,\theta) \tag{33}$$

Equation (33) may be solved by an exponential approach with $t_0 := (t=0)$.

$$\hat{p}(t,\omega,\theta) = \hat{p}(t_0,\omega,\theta)e^{-D(\omega^2+\theta^2)t} \tag{34}$$

here $p(t_0,x,y)$ is defined as:

$$p(t_0,x,y) = p_o\delta^2(x,y) \tag{35}$$

With δ representing the delta-function equation (35) can be solved to:

$$\begin{aligned}\hat{p}(t_0,\omega,\theta) &= \mathscr{F}\{p(t_0,x,y)\}\\ &= \int_{-\infty}^{\infty}\int_{-\infty}^{\infty} e^{i(\omega x+\theta y)}p_0\delta^2(x,y)\,dx\,dy\\ &= p_0\end{aligned} \tag{36}$$

backward fourier transformation of $\hat{p}(t,\omega,\theta)$ results in:

$$\begin{aligned}p(t,x,y) &= \mathscr{F}^{-1}\{\hat{p}(t,x,y)\}\\ &= \frac{1}{(2\pi)^2}\int_{-\infty}^{\infty}\int_{-\infty}^{\infty} e^{-i(\omega x+\theta y)}\\ &\quad p_0e^{-D(\omega^2+\theta^2)t}\,d\omega\,d\theta\end{aligned} \tag{37}$$

extending the exponents by $\pm\frac{x^2}{4Dt}$ results to:

$$\begin{aligned}&e^{-i(\omega x+\theta y)}e^{-D(\omega^2+\theta^2)t}\\ &= e^{-(i\omega x+D\omega^2t)}e^{-(i\theta y+D\theta^2t)}\\ &= e^{-\left(\frac{-x^2}{4Dt}+i\omega x+D\omega^2t+\frac{x^2}{4Dt}\right)}e^{-\left(\frac{-y^2}{4Dt}+i\theta y+D\theta^2t+\frac{y^2}{4Dt}\right)}\\ &= e^{-\left(\frac{ix}{2\sqrt{Dt}}+\omega\sqrt{Dt}\right)^2}e^{\frac{-x^2}{4Dt}}e^{-\left(\frac{iy}{2\sqrt{Dt}}+\theta\sqrt{Dt}\right)^2}e^{\frac{-y^2}{4Dt}}\end{aligned} \tag{38}$$

Insert in equation (37) results in:

$$\begin{aligned}p(t,x,y) &= \frac{1}{(2\pi)^2}\int_{-\infty}^{\infty}\int_{-\infty}^{\infty} e^{-\left(\frac{ix}{2\sqrt{Dt}}+\omega\sqrt{Dt}\right)^2}e^{\frac{-x^2}{4Dt}}\\ &\quad e^{-\left(\frac{iy}{2\sqrt{Dt}}+\theta\sqrt{Dt}\right)^2}e^{\frac{-y^2}{4Dt}}p_0\,d\omega\,d\theta\\ &= \frac{p_0}{(2\pi)^2}e^{\frac{-(x^2+y^2)}{4Dt}}\\ &\quad\int_{-\infty}^{\infty}\int_{-\infty}^{\infty} e^{-\left(\frac{ix}{2\sqrt{Dt}}+\omega\sqrt{Dt}\right)^2}\\ &\quad e^{-\left(\frac{iy}{2\sqrt{Dt}}+\theta\sqrt{Dt}\right)^2}\,d\omega\,d\theta\end{aligned} \tag{39}$$

Which can be further simplified by the variants of constants:

$$\frac{ix}{2\sqrt{Dt}} + \omega\sqrt{Dt} = \Omega \tag{40}$$

$$\frac{\partial\Omega}{\partial\omega} = \sqrt{Dt} \tag{41}$$

$$d\omega = d\Omega\frac{1}{\sqrt{Dt}} \tag{42}$$

$$\frac{iy}{2\sqrt{Dt}} + \theta\sqrt{Dt} = \Theta \tag{43}$$

$$\frac{\partial\Theta}{\partial\theta} = \sqrt{Dt} \tag{44}$$

$$d\theta = d\Theta\frac{1}{\sqrt{Dt}} \tag{45}$$

So finally we end up with:

$$
\begin{aligned}
p(t,x,y) &= \frac{p_0}{(2\pi)^2} e^{\frac{-(x^2+y^2)}{4Dt}} \\
&\int_{-\infty}^{\infty}\int_{-\infty}^{\infty} e^{-\Omega^2} e^{-\Theta^2} \frac{1}{Dt}\, d\Omega\, d\Theta \\
&= \frac{p_0}{4\pi Dt} e^{-\frac{x^2+y^2}{4Dt}} \\
&= \frac{1}{4\pi Dt} e^{-\frac{x^2+y^2}{4Dt}}
\end{aligned} \tag{46}
$$

Equation (46) has an expectation value of:

$$
\begin{aligned}
\langle r^2 \rangle &= \int_{-\infty}^{\infty}\int_{-\infty}^{\infty} (x^2+y^2) p(t,x,y)\, dx\, dy \\
&= \frac{1}{4\pi Dt} \int_{-\infty}^{\infty}\int_{-\infty}^{\infty} (x^2+y^2) e^{\frac{-(x^2+y^2)}{4Dt}}\, dx\, dy \\
&= 4Dt
\end{aligned} \tag{47}
$$

As we want to address only point-to-point distances, we can further simplify equation (46) by a transformation into polar coordinates ($r = \sqrt{x^2+y^2}$) and integrate over the line element ($ds_\phi = r\, d\phi$).

$$
\begin{aligned}
p(t,r,\phi) &= \frac{1}{4\pi Dt} \int_0^{2\pi} r e^{\frac{-(r^2)}{4Dt}}\, d\phi \\
&= \frac{2r}{4Dt} e^{\frac{-(r^2)}{4Dt}}
\end{aligned} \tag{48}
$$

In combination with the expectation value (see equation (47)) we get:

$$
p(t,r) = \frac{2r}{\langle r^2 \rangle} e^{\frac{-(r^2)}{\langle r^2 \rangle}} \tag{49}
$$

Lets assume that each fluorophore within our measurement is detected in two adjacent frames (which is feasible because the fluorophores' blinking rates are not in phase with the acquisition frame rate). This translates into two populations of fluorophores: The first population is detected in the first of the two frames at time t and the second population is detected in the adjacent frame $t+1$. Each localization pair that is occurring from the same fluorophore represents a single point-to-point distance (from two points belonging to two different localization distributions). As we have only one sort of fluorophore within our experiment from which we would expect the same photo-physical behavior, we could pool all of these distances. We end up with a distribution of point-to-point distances from localizations between two Gaussian-shaped (see equation 50) localization distributions.

$$
p(x) = \frac{1}{\sqrt{2\pi\sigma^2}} e^{-\frac{(x-\mu)^2}{2\sigma^2}} \tag{50}
$$

Because we have only measured inter-distribution point-to-point distances we do not know the actual center of both distributions. A method to analyze the mean center-to-center distance from these kind of data was developed by Churchman et al. [2005] and shall be described briefly. Lets assume that both distributions are separated by an unknown distance μ. In order to simplify the math of the problem we will rotate the coordinate system in a way that the two functions are only separated in one dimension by a distance of $\Delta x = \mu$:

$$
\begin{aligned}
\langle d^2 \rangle = &\int_{-\infty}^{\infty}\int_{-\infty}^{\infty}\int_{-\infty}^{\infty}\int_{-\infty}^{\infty} d^2 \\
&p(x_1)p(x_2)p(y_1)p(y_2)\, dx_1\, dx_2\, dy_1\, dy_2
\end{aligned} \tag{51}
$$

with $d^2 = \left((x_1-x_2)^2 + (y_1-y_2)^2\right)$ and:

$$\mu_{x1} = 0 \tag{52}$$
$$\mu_{x2} = \mu \tag{53}$$
$$\mu_{y1} = 0 \tag{54}$$
$$\mu_{y2} = 0 \tag{55}$$

Equatition (51) can be solved with:

$$\int_{-\infty}^{\infty} x^2 p(x)\, dx = \sigma^2 \tag{56}$$
$$\int_{-\infty}^{\infty} x p(x)\, dx = \mu \tag{57}$$
$$\int_{-\infty}^{\infty} p(x)\, dx = 1 \tag{58}$$

to:

$$
\begin{aligned}
\langle d^2 \rangle = &\int_{-\infty}^{\infty}\int_{-\infty}^{\infty}\int_{-\infty}^{\infty}\int_{-\infty}^{\infty} \\
&\Big(x_1^2 + 2x_1x_2 + (x_2-\mu)^2 \\
&+ 2x_2\mu - \mu^2 + y_1^2 - 2y_1y_2 + y_2^2\Big) \\
&\frac{1}{\sqrt{2\pi\sigma_1^2}} e^{-\frac{x_1^2}{2\sigma_1^2}} \\
&\frac{1}{\sqrt{2\pi\sigma_2^2}} e^{-\frac{(x_2-\mu)^2}{2\sigma_2^2}} \\
&\frac{1}{\sqrt{2\pi\sigma_1^2}} e^{-\frac{y_1^2}{2\sigma_1^2}} \\
&\frac{1}{\sqrt{2\pi\sigma_2^2}} e^{-\frac{y_2^2}{2\sigma_2^2}}\, dx_1\, dx_2\, dy_1\, dy_2
\end{aligned} \tag{59}
$$

$$\langle d^2 \rangle = \mu^2 + 2(\sigma_1^2 + \sigma_2^2) \tag{60}$$

For a point-symmetric Gaussian distribution, we can assume: $\sigma_1 = \sigma_2 = \sigma_r$

$$\langle d^2 \rangle = \mu^2 + 4\sigma^2 \tag{61}$$

For a the distance distribution between two point-symmetric probability functions from the type of equation (50) we get:

$$
p(t,d) = \frac{2D}{\mu(t)^2 + 4\sigma^2} e^{-\frac{d^2}{\mu(t)^2+4\sigma^2}} \tag{62}
$$

Insertion of equation (47) leeds to:

$$p(t,d) = \frac{2D}{4Dt + 4\sigma^2} e^{-\frac{d^2}{4Dt+4\sigma^2}} \tag{63}$$

For a fixed sample the time frozen to t_0 ($\Delta t = 0$) and therefore an expectation value of $\mu(\Delta t) = 4D\Delta t = 0$ can be assumed. We end up with a probability density function of the experimental localization precision.

$$p(d) = \frac{2d}{4\sigma^2} e^{-\frac{d^2}{4\sigma^2}} \tag{64}$$

Equation (64) allows the calculation of the localization precision from the distribution of point-to-point distances of multiple localization routines.

B.2 Edge correction for Ripley's K-function

Possible methods of edge correction are the introduction of a buffer zone, the calculation of a passepartout containing a continuing localization pattern, or the adaption of the detected neighbors $N_r(d_{ij})$ by a weighting factor ω to the effectively observed neighboring area [Haase, 1995].

B.2.1 Buffer Zones

The introduction of a buffer zone is based on decoupling the pool of investigated localizations i located in $\mathrm{ROI_i}$ from the pool of possible neighboring localizations j located in $\mathrm{ROI_j}$ [Sterner et al., 1986]. By enlarging the area of $\mathrm{ROI_j}$ in comparison to the area of $\mathrm{ROI_i}$ a buffer zone is introduced containing additional localizations. In order to correct for edge effects of a K-function with a maximal observed neighborhood distance $r_{\max}$, $\mathrm{ROI_j}$ has to guarantee that for every investigated localization i (even if located exactly at the edge of $\mathrm{ROI_i}$) the full area of possible neighbors at $r_{\max}$ is present in the pool of neighboring localizations j. For squared ROIs, the area A_j of $\mathrm{ROI_j}$ is related to the area A_i of $\mathrm{ROI_i}$ defined by an edge length of a_i by:

$$A_j = (a_i + 2r_{\max})^2 \tag{65}$$

This method of edge correction describes a pattern in the most realistic way as no assumption or annealing has to be calculated. The main drawback of introducing a buffer zone is the limitation in ROI selection; The protein distribution within a biological cell for example is spatially limited to the cell membrane. Buffer zones cannot guarantee a satisfactory correction for edge effects if the $\mathrm{ROI_i}$ is located at the edge of the cell. $\mathrm{ROI_i}$ has to be dislocated at least by a distance of r_{max} from all edges of the cell membrane. This may hinder the investigation of larger $\mathrm{ROI_i}$. A minor drawback of a buffer zone is that an increasing pool of possible neighbor localizations j also increases the computing time.

B.2.2 Weighting Factor

This method works on a $\mathrm{ROI_i}$ identically to $\mathrm{ROI_j}$, which gives two identical pools i and j of investigated localizations and possible neighbors. If the area of the investigated neighborhood exceeds the boundaries of the ROI, the introduction of a weighting factor ω may correct the detected neighbors $N_r(d_{ij})$ out of j around a localization i within a distance r for the effectively observed area Getis and Franklin [1987], Andersen [1992], Haase [1995]. ω is based upon the assumption that the distribution pattern outside the ROI boundaries is exactly the same as inside. ω has to be considered for four different cases.

If the observed distance r around i is smaller than the distance to the nearest ROI boundary, no additional weighting is necessary as all possible neighbors are existing within j.

If the observed distance r around i is larger than the distance to the nearest ROI boundary b_1, ω calculates to:

$$\omega_r = 1 - \frac{\cos^{-1}(\frac{b_1}{r})}{\pi} \tag{66}$$

If the observed distance r around i is larger than the distance to two ROI boundaries b_1 and b_2, ω calculates to:

$$\omega_r = 1 - \left[\cos^{-1}\left(\frac{b_1}{r}\right) + \cos^{-1}\left(\frac{b_2}{r}\right) + \frac{\pi}{2}\right] 2\pi \tag{67}$$

If the observed distance r around i is larger than the distance to two ROI boundaries b_1 and b_2 and r is smaller than the distance between i and the nearest corner, ω calculates to:

$$\omega_r = 1 - \left[2\cos^{-1}\left(\frac{b_1}{r}\right) + 2\cos^{-1}\left(\frac{b_2}{r}\right)\right] 2\pi \tag{68}$$

A main advantage of this technique is that the ROI can be chosen freely, as $\mathrm{ROI_i}$ is identical to $\mathrm{ROI_j}$. An additional advantage is the fact that the pool of possible neighbors j has not to be enlarged as in the case of a buffer zone introduction. A disadvantage is the assumption that the localization pattern within the complete distance r around i is of a similar distribution, which is only true for a uniform distribution. Correcting for edge effects via a weighting factor is the method of choice in samples exhibiting localization patterns near a uniform distribution but it may lead to a general underestimation of the degree of clustering in highly clustered samples. This effect enlarges with the size of the neighborhood that has to be assumed.

B.2.3 Toroidal Edge Correction

The method of toroidal edge correction combines the two methods described above. For toroidal edge correction the degree of clustering is assumed to be consistent within a large area around the ROI. The distribution of investigated localizations i within the ROI is projected onto a three-dimensional surface with no existing ROI boundaries. As a result, localizations that were located at opposing boundaries before are now located in close neighborhood (see figure 27). Toroidal edge correction is realized by computing a passepartout consisting of 8 identical copies of the ROI around the original ROI [Haase, 1995]. This passepartout serves as an annealed

buffer zone. As toroidal edge correction enlarges the pool of possible neighbors j, it suffers from the same need of high *Central Processing Unit* (CPU) power as the buffer zone method. It gives the user the opportunity to freely choose the ROI. It creates a fictional buffer zone that might as well as the weighting factor lead to misinterpretations on the degree of clustering.

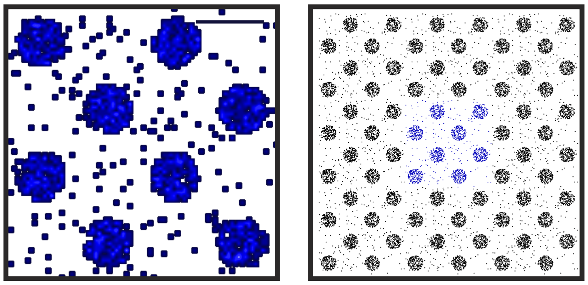

Figure 27. Toroidal edge correction
Simulation of single-molecule localization data for the analysis with Ripley's K-function. Single-molecule localization data of clusters with a diameter of 140 nm were simulated and background noise was added. The clusters were spaced in a regular pattern with the same average distance (scale bar: 200 nm). For cluster analysis using the K-function, the simulated data were extended to a 3 x 3 area to minimize edge effects (Graph adopted from Malkusch et al. [2013]).

B.3 On the use of Markov chains for the analysis of blinking kinetics at the single molecule level

Analyzing the oligomeric state of reversible photoswitching fluorophores at the single-molecule level builds on a simple model: a fluorophore is existing in two states (an active state A and a photo-bleached state B) is bleaching over time with a fixed reaction rate constant k (see figure 28).

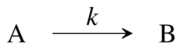

Figure 28. Kinetic scheme for fluorescence photobleaching

The detected fluorescence intensity is directly proportional to the concentration of flurorophores in the active state $[A]$. For a first order photobleaching reaction the decrease in intensity is described by a mono-exponential decay (see equation (70)).

$$\frac{d[A]}{dt} = -k[A] \tag{69}$$

$$[A] = [A]_0\, e^{-kt} \tag{70}$$

At the single-molecule level the reaction is described by a *Markov process*. In case of a mono-exponential decay of fluorescence, the probability distribution P(X=0, $[t_0, t]$) describes that after a period of time between $t_0 = 0$ and t ($[t_0, t]$) no reaction took place ($X = 0$), which means that the molecule still exists in state A (see equation (71)) [Levitus, 2010].

$$\begin{aligned} P(X = 0, [t_0, t]) &= \frac{[A]}{[A]_0} \\ &= e^{-kt} \end{aligned} \tag{71}$$

From equation (71) one can derive the probability distribution P(X=1, $[t_0, t]$), which describes that a single molecule undergoes a reaction within the time period $[t_0, t]$, which means that a single photobleaching event took place (X=1).

$$\begin{aligned} P(X = 1, [t_0, t]) &= 1 - P(X = 0, [t_0, t]) \\ &= 1 - e^{-kt} \end{aligned} \tag{72}$$

The probability density distribution p(X=1,t) of the differentiable probability distribution P(X=1, $[t_0, t]$), describes the probability of an event happening exactly at time point t, and is given by the derivation of equation (72).

$$\begin{aligned} p(X = 1, t) &= \frac{d}{dt} P(X = 1, [t_0, t]) \\ &= k\, e^{-kt} \end{aligned} \tag{73}$$

In reality, the photophysics of ps-FPs at the single molecule level are more complicated. A valid approximation is the description by a minimal photo-kinetic 4-state model (see figure 29) [Lee et al., 2012].

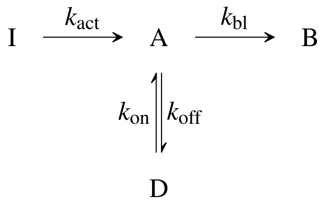

Figure 29. A minimal switching model
The fluorophore is activated from an inactive state I to the active state A with k_{act}. State A is characterized by fluorescence emission. From state A the flourophore can either bleach irreversibly to the bleached state B with k_{bl}, or it can reversibly switch to a dark state D with k_{off}. The reversibly switching from D to A accurs with k_{on}.

The initial state of the fluorophore is the inactive state I. It is transferred into the active state A with the activation rate constant k_{act}. A fluorophore in the active state emits fluorescence. It is therefore the only detected state during an SMLM experiment. From state A the fluorophore can be transferred into the bleached state B by the bleaching rate constant k_{bl}. Alternatively, the fluorophore can switch between state A and a dark state D by a reversible reaction described by the reaction rate constant k_{off} and vice versa by k_{on}.
In order to describe the blinking kinetics, the technical term *blinking* needs to be defined: *blinking* is the return of the fluorophore from the dark state D to the active state A. From the experimental view, *blinking* is the recurrence of fluorescence.

A typical intensity time trace of a fluorophore that undergoes a single blinking event X_D before photo bleaching X_B is depicted in figure 30. The fluorophore is activated from state I to state A. As long as the fluorophore exists in state A, fluorescence is detected. The time period the fluorophore stays in state A is termed t_{on}. The fluorophore switches reversibly between the dark state D and A. The time period the fluorophore exists in state D is termed t_{off}. This procedure of fluorescence reoccurrence is termed a blinking event. For each blinking event the fluorophore perfomes, the counter for blinking is increased by 1 (in case of figure 30: $X_D = 1$). The time period between the activation event and the photobleaching event, or the time the fluorophore is switching between state A and D, is termed t.

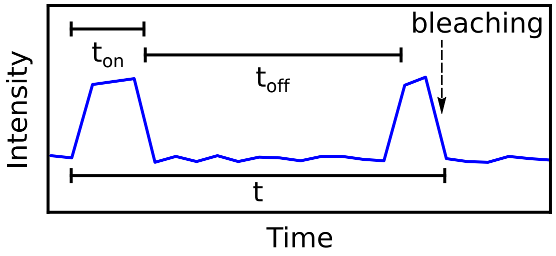

Figure 30. Intensity time trace of a single fluorophore
The fluorophore is activated for t_{on} in state A. It switches spontaneously to state D and stays for t_{off} in the dark state until it switches back to state A. It undergoes a bleaching process into state B and is no longer detected. As the fluorophore recovers once from the dark state D to the active state A before photobleaching, it performs a single blinking event $X_D = 1$. The time t between the activation of the fluorophore and its photobleaching is termed the blinking period.

The most simple reaction chain for an active molecule until photobleaching ($X_B = 1$) is without any blinking event ($X_D = 0$). The probability density distribution of a single molecule, that is transferred to the active state at t_0 and bleaches at time point t without undergoing a single event of blinking p($X_\mathrm{D} = 0$, $X_\mathrm{B} = 1$, t) can be derived from equations (71) and (73):

$$\begin{aligned} &p(X_\mathrm{D} = 0, X_\mathrm{B} = 1, t) \\ &= P_{k_\mathrm{off}}(X_D = 0, [t_0, t])\; p_{k_\mathrm{bl}}(X_B = 1, t) \\ &= e^{-k_\mathrm{off} t}\; k_\mathrm{bl}\; e^{-k_\mathrm{bl} t} \end{aligned} \tag{74}$$

The probability to see no blinking at all is therefore described by:

$$\begin{aligned} &P(X_\mathrm{D} = 0, X_\mathrm{B} = 1, [t_0, \infty]) \\ &= \int_0^\infty e^{-k_\mathrm{off} t}\; k_\mathrm{bl}\; e^{-k_\mathrm{bl} t} dt \\ &= \int_0^\infty k_\mathrm{bl}\; e^{-t(k_\mathrm{bl} + k_\mathrm{off})} dt \\ &= \frac{k_\mathrm{bl}}{k_\mathrm{off} + k_\mathrm{bl}} \end{aligned} \tag{75}$$

The probability density distribution for a molecule that undergoes exactly one blinking event ($X_D = 1$) until photobleaching ($X_B = 1$) at time point t (p($X_D = 1$, $X_B = 1$, t)) calculates to:

$$\begin{aligned} &p(X_\mathrm{D} = 1, X_\mathrm{B} = 1, t) = \\ &\int_0^t dt_\mathrm{on}\; p_{k\mathrm{off}}(X_\mathrm{D} = 1, t_\mathrm{on})\; P_{k_\mathrm{bl}}(X_\mathrm{B} = 0, [t_0, t_\mathrm{on}]) * \\ &\int_0^{t - t_\mathrm{on}} dt_\mathrm{off}\; p_{k_\mathrm{on}}(X_\mathrm{A} = 1, t_\mathrm{off}) * \\ &(P_{k_\mathrm{off}}(X_\mathrm{D} = 0, [t_0, t - t_\mathrm{on} - t_\mathrm{off}]) * \\ &p_{k_\mathrm{bl}}(X_\mathrm{bl} = 1, t - t_\mathrm{on} - t_\mathrm{off})) \end{aligned} \tag{76}$$

With

$$p_1(t) = p_{k\mathrm{off}}(X_\mathrm{D} = 1, t)\; P_{k_\mathrm{bl}}(X_\mathrm{B} = 0, [t_0, t]) \tag{77}$$

$$p_2(t) = p_{k_\mathrm{on}}(X_\mathrm{A} = 1, t) \tag{78}$$

$$p_3(t) = P_{k_\mathrm{off}}(X_\mathrm{D} = 0, [t_0, t])\; p_{k_\mathrm{bl}}(X_\mathrm{bl} = 1, t) \tag{79}$$

equation (76) can be simplified to:

$$\begin{aligned} &p(X_\mathrm{D} = 1, X_\mathrm{B} = 1, t) = \\ &\int_0^t dt_\mathrm{on}\; p_1(t_\mathrm{on}) \int_0^{t - t_\mathrm{on}} dt_\mathrm{off}\; p_2(t_\mathrm{off})\; p_3(t - t_\mathrm{on} - t_\mathrm{off}) \end{aligned} \tag{80}$$

With p_1 being the probability of a transition to the dark state, p_2 being the probability of a transition from the dark state to the active state, and p_3 being the probability of bleaching event with no further blinking reactions.
Considering the definition of a convolution:

$$\int_0^t f(\tau)\; g(t - \tau) d\tau = f \otimes g \tag{81}$$

equation (80) can be redefined to:

$$p(X_\mathrm{D} = 1, X_\mathrm{B} = 1, t) = (p_1 \otimes p_2 \otimes p_3(t)) \tag{82}$$

The probability of recording a distinct number (n) of blinking events ($X_\mathrm{D} = n$) before photobleaching ($X_\mathrm{B} = 1$) at time point t from a single ps-FP is therefore given by:

$$\begin{aligned} &p(X_\mathrm{D} = n, X_\mathrm{B} = 1, t) = \\ &(p_1 \otimes p_2)_1 \otimes \ldots \otimes (p_1 \otimes p_2)_n \otimes p_3(t) \end{aligned} \tag{83}$$

Equation (83) can easily be solved by a Laplace transformation [Lee et al., 2012], which is defined as follows:

$$\begin{aligned} \tilde{a}(s) &= \mathscr{L}\{A\}(s) \\ &= \int_0^\infty a(t)\; e^{-st}\; dt \end{aligned} \tag{84}$$

Laplace transformation of p_1, p_2, and p_3 calculates to:

$$\begin{aligned}\tilde{p}_1(s) &= \int_0^\infty k_{\text{off}}\, e^{-t(k_{\text{off}}+k_{\text{bl}}+s)}\, dt \\ &= \frac{k_{\text{off}}}{k_{\text{off}}+k_{\text{bl}}+s}\end{aligned} \tag{85}$$

$$\begin{aligned}\tilde{p}_2(s) &= \int_0^\infty k_{\text{on}}\, e^{-t(k_{\text{on}}+s)}\, dt \\ &= \frac{k_{\text{on}}}{k_{\text{on}}+s}\end{aligned} \tag{86}$$

$$\begin{aligned}\tilde{p}_3(s) &= \int_0^\infty k_{\text{bl}}\, e^{-t(k_{\text{off}}+k_{\text{bl}}+s)}\, dt \\ &= \frac{k_{\text{bl}}}{k_{\text{off}}+k_{\text{bl}}+s}\end{aligned} \tag{87}$$

The convolution theorem of Laplace,

$$\int_0^t f(\tau)\, g(t-\tau)\, dt = \tilde{f}(s)\, \tilde{g}(s) \tag{88}$$

states, that a convolution in Laplace space is turned into a multiplication. Therefore, equation (83) in Laplace space calculates to:

$$\begin{aligned}&\tilde{p}(X_{\text{D}}=n, X_{\text{B}}=1, s) = (\tilde{p}_1\, \tilde{p}_2)^n\, \tilde{p}_3 \\ &= \left(\frac{k_{\text{off}}}{k_{\text{off}}+k_{\text{bl}}+s}\, \frac{k_{\text{on}}}{k_{\text{on}}+s}\right)^n \frac{k_{\text{bl}}}{k_{\text{off}}+k_{\text{bl}}+s}\end{aligned} \tag{89}$$

In the case of $s=0$ the Laplace transformation of the probability density distribution $\tilde{p}(s=0)$ turns into the probability distribution P:

$$\begin{aligned}\tilde{p}(s=0) &= \int_0^\infty p(t)\, e^{-st}\, dt \\ &= \int_0^\infty p(t)\, dt \\ &= P([0,\infty])\end{aligned} \tag{90}$$

Therefore, the probability for n blinking events calculates from equation (89) to:

$$\begin{aligned}&P(X_{\text{D}}=n, X_{\text{B}}=1, [0,\infty]) = \\ &\tilde{p}(X_{\text{D}}=n, X_{\text{B}}=1, s=0) = \\ &= \left(\frac{k_{\text{off}}}{k_{\text{off}}+k_{\text{bl}}}\right)^n \frac{k_{\text{bl}}}{k_{\text{off}}+k_{\text{bl}}} \\ &= \left(1-\frac{k_{\text{bl}}}{k_{\text{off}}+k_{\text{bl}}}\right)^n \frac{k_{\text{bl}}}{k_{\text{off}}+k_{\text{bl}}}\end{aligned} \tag{91}$$

The probability of detecting n blinking events (short P_n), given by equation (91), can be simplified by inserting the probability $P(X_{\text{D}}=0, X_{\text{B}}=1, [0,\infty])$ for detecting no blinking event from equation (75) (short P_0) to:

$$\begin{aligned}P_n &= \left(1-\frac{k_{\text{bl}}}{k_{\text{off}}+k_{\text{bl}}}\right)^n \frac{k_{\text{bl}}}{k_{\text{off}}+k_{\text{bl}}} \\ &= (1-P_0)^n\, P_0\end{aligned} \tag{92}$$

Acronyms

ad *analog-to-digital*

bcm *binary cluster map*

CBC *coordinate based colocalization*

CPU *Central Processing Unit*

DBSCAN *Density-Based Algorithm for Discovering Clusters in Large Spatial Databases with Noise*

EMCCD *Electron Multiplying Charged Coupled Device*

FAS *focal adhesion site*

FP *fluorescent protein*

FRC *Fourier ring correlation*

FRET *Foerster Resonance Energy Transfer*

GNU *GNU is Not Unix*

GPL *GNU publishing license*

GUI *graphical user interface*

HIV *human immunodeficiency virus*

Lama *LocAlization Microscopy Analyzer*

LSE *least-squares estimation*

lwm *local weight matrix*

Malk *Molecular Accuracy Localization Keep*

MCA *Morphological Cluster Analysis*

MLE *maximum likelihood estimation*

NeNA *Nearest Neighbor in Adjacent frames*

OPTICS *Ordering Points To Identify the Clustering Structure*

PALM *photo activated localization microscopy*

pixel *picture element*

ps *photo-switchable*

PSF *point spread function*

PSFs *point spread functions*

rapidSTORM *rapid yet accurate program implementing the direct stochastic optical reconstruction microscopy*

ROI *region of interest*

SM *single molecule*

SMB *Single Molecule Biophysics*

SMLM *single-molecule localization microscopy*

SNR *signal to noise ratio*

STORM *stochastic optical reconstruction microscopy*

TNFR1 *tumor necrosis factor receptor 1*

References

Ernst Abbe. Beiträge zur Theorie des Mikroskops und der mikroskopischen Wahrnehmung. *Archiv für mikroskopische Anatomie*, 9(1):413–418, 1873.

Mark Andersen. Spatial analysis of two-species interactions. *Oecologia*, 91(1):134–140, 1992.

Leonid Andronov, Yves Lutz, Jean-Luc Vonesch, and Bruno P Klaholz. Sharpvisu: integrated analysis and segmentation of super-resolution microscopy data. *Bioinformatics*, page btw123, 2016a.

Leonid Andronov, Igor Orlov, Yves Lutz, Jean-Luc Vonesch, and Bruno P Klaholz. Clustervisu, a method for clustering of protein complexes by voronoi tessellation in super-resolution microscopy. *Scientific reports*, 6, 2016b.

Mihael Ankerst, Markus M Breunig, Hans-Peter Kriegel, and Jörg Sander. Optics: ordering points to identify the clustering structure. In *ACM Sigmod Record*, volume 28, pages 49–60. ACM, 1999.

Sergiy Avilov, Romain Berardozzi, Mudalige S Gunewardene, Virgile Adam, Samuel T Hess, and Dominique Bourgeois. In cellulo evaluation of phototransformation quantum yields in fluorescent proteins used as markers for single-molecule localization microscopy. *PloS one*, 9(6):e98362, 2014.

C Bradford Barber, David P Dobkin, and Hannu Huhdanpaa. The quickhull algorithm for convex hulls. *ACM Transactions on Mathematical Software (TOMS)*, 22(4): 469–483, 1996.

Anthony Barsic, Ginni Grover, and Rafael Piestun. Three-dimensional super-resolution and localization of dense clusters of single molecules. *Scientific reports*, 4, 2014.

Eric Betzig, George H Patterson, Rachid Sougrat, O Wolf Lindwasser, Scott Olenych, Juan S Bonifacino, Michael W Davidson, Jennifer Lippincott-Schwartz, and Harald F Hess. Imaging intracellular fluorescent proteins at nanometer resolution. *Science*, 313(5793):1642–1645, 2006.

S Bolte and FP Cordelieres. A guided tour into subcellular colocalization analysis in light microscopy. *Journal of microscopy*, 224(3):213–232, 2006.

John AG Briggs, James D Riches, B Glass, V Bartonova, Giulia Zanetti, and H-G Kräusslich. Structure and assembly of immature hiv. *Proceedings of the National Academy of Sciences*, 106(27):11090–11095, 2009.

Fabiana A Caetano, Brennan S Dirk, Joshua HK Tam, P Craig Cavanagh, Maria Goiko, Stephen SG Ferguson, Stephen H Pasternak, Jimmy D Dikeakos, John R de Bruyn, and Bryan Heit. Miisr: Molecular interactions in super-resolution imaging enables the analysis of protein interactions, dynamics and formation of multi-protein structures. *PLoS Comput Biol*, 11(12):e1004634, 2015.

L Stirling Churchman, Zeynep Ökten, Ronald S Rock, John F Dawson, and James A Spudich. Single molecule high-resolution colocalization of cy3 and cy5 attached to macromolecules measures intramolecular distances through time. *Proceedings of the National Academy of Sciences of the United States of America*, 102(5):1419–1423, 2005.

Genome Damage and Stability Centre at the University of Sussex. URL `http://www.sussex.ac.uk/gdsc/intranet/microscopy/imagej/smlm_plugins`.

H Deschout, A Shivanandan, P Annibale, M Scarselli, and A Radenovic. Progress in quantitative single-molecule localization microscopy. *Histochemistry and cell biology*, 142(1):5–17, 2014a.

Hendrik Deschout, Francesca Cella Zanacchi, Michael Mlodzianoski, Alberto Diaspro, Joerg Bewersdorf, Samuel T Hess, and Kevin Braeckmans. Precisely and accurately localizing single emitters in fluorescence microscopy. *Nature methods*, 11(3):253–266, 2014b.

Laura Tarancón Díez, Claudia Bönsch, Sebastian Malkusch, Zinnia Truan, Mihaela Munteanu, Mike Heilemann, Oliver Hartley, Ulrike Endesfelder, and Alexandre Fürstenberg. Coordinate-based co-localization-mediated analysis of arrestin clustering upon stimulation of the c–c chemokine receptor 5 with rantes/ccl5 analogues. *Histochemistry and cell biology*, pages 1–9, 2014.

Mohamed El Beheiry and Maxime Dahan. Visp: representing single-particle localizations in three dimensions. *Nature methods*, 10(8):689–690, 2013.

Ulrike Endesfelder, Sebastian Malkusch, Benjamin Flottmann, Justine Mondry, Piotr Liguzinski, Peter J Verveer, and Mike Heilemann. Chemically induced photoswitching of fluorescent probes—a general concept for super-resolution microscopy. *Molecules*, 16(4):3106–3118, 2011.

Ulrike Endesfelder, Sebastian Malkusch, Franziska Fricke, and Mike Heilemann. A simple method to estimate the average localization precision of a single-molecule localization microscopy experiment. *Histochemistry and cell biology*, 141(6):629–638, 2014.

Martin Ester, Hans-Peter Kriegel, Jörg Sander, and Xiaowei Xu. A density-based algorithm for discovering clusters in large spatial databases with noise. In *Kdd*, volume 96, pages 226–231, 1996.

Jonas Fölling, Mariano Bossi, Hannes Bock, Rebecca Medda, Christian A Wurm, Birka Hein, Stefan Jakobs, Christian Eggeling, and Stefan W Hell. Fluorescence nanoscopy by ground-state depletion and single-molecule return. *Nature Methods*, 5(11):943–945, 2008.

Franziska Fricke, Sebastian Malkusch, Gaby Wangorsch, Johannes F Greiner, Barbara Kaltschmidt, Christian Kaltschmidt, Darius Widera, Thomas Dandekar, and Mike Heilemann. Quantitative single-molecule localization microscopy combined with rule-based modeling reveals ligand-induced tnf-r1 reorganization toward higher-order oligomers. *Histochemistry and cell biology*, pages 1–11, 2014.

Franziska Fricke, Joel Beaudouin, Roland Eils, and Mike Heilemann. One, two or three? probing the stoichiometry of membrane proteins by single-molecule localization microscopy. *Scientific Reports*, 2015.

Alexandre Fürstenberg and Mike Heilemann. Single-molecule localization microscopy–near-molecular spatial resolution in light microscopy with photoswitchable fluorophores. *Physical Chemistry Chemical Physics*, 15(36):14919–14930, 2013.

Arthur Getis and Janet Franklin. Second-order neighborhood analysis of mapped point patterns. *Ecology*, 68(3):473–477, 1987.

Peter Haase. Spatial pattern analysis in ecology based on Ripley's K-function: Introduction and methods of edge correction. *Journal of Vegetation Science*, 6(4):575–582, 1995.

Mike Heilemann, Sebastian van de Linde, Mark Schüttpelz, Robert Kasper, Britta Seefeldt, Anindita Mukherjee, Philip Tinnefeld, and Markus Sauer. Subdiffraction-resolution fluorescence imaging with conventional fluorescent probes. *Angewandte Chemie International Edition*, 47(33):6172–6176, 2008.

Ricardo Henriques, Mickael Lelek, Eugenio F Fornasiero, Flavia Valtorta, Christophe Zimmer, and Musa M Mhlanga. Quickpalm: 3d real-time photoactivation nanoscopy image processing in imagej. *Nature methods*, 7(5):339–340, 2010.

Bo Huang, Wenqin Wang, Mark Bates, and Xiaowei Zhuang. Three-dimensional super-resolution imaging by stochastic optical reconstruction microscopy. *Science*, 319 (5864):810–813, 2008.

Manuel F Juette, Travis J Gould, Mark D Lessard, Michael J Mlodzianoski, Bhupendra S Nagpure, Brian T Bennett, Samuel T Hess, and Joerg Bewersdorf. Three-dimensional sub–100 nm resolution fluorescence microscopy of thick samples. *Nature methods*, 5(6):527–529, 2008.

Adel Kechkar, Deepak Nair, Mike Heilemann, Daniel Choquet, and Jean-Baptiste Sibarita. Real-time analysis and visualization for single-molecule based super-resolution microscopy. *PLoS One*, 8(4):e62918, 2013.

Ullrich Köthe, Frank Herrmannsdörfer, Ilia Kats, and Fred A Hamprecht. Simplestorm: a fast, self-calibrating reconstruction algorithm for localization microscopy. *Histochemistry and cell biology*, 141(6):613–627, 2014.

Sang-Hyuk Lee, Jae Yen Shin, Antony Lee, and Carlos Bustamante. Counting single photoactivatable fluorescent molecules by photoactivated localization microscopy (palm). *Proceedings of the National Academy of Sciences*, 109(43):17436–17441, 2012.

Florian Levet, Eric Hosy, Adel Kechkar, Corey Butler, Anne Beghin, Daniel Choquet, and Jean-Baptiste Sibarita. Sr-tesseler: a method to segment and quantify localization-based super-resolution microscopy data. *Nature methods*, 12(11):1065–1071, 2015.

Marcia Levitus. Chemical kinetics at the single-molecule level. *Journal of Chemical Education*, 88(2):162–166, 2010.

Björn F Lillemeier, Manuel A Mörtelmaier, Martin B Forstner, Johannes B Huppa, Jay T Groves, and Mark M Davis. Tcr and lat are expressed on separate protein islands on t cell membranes and concatenate during activation. *Nature immunology*, 11(1):90–96, 2010.

Sebastian Malkusch, Ulrike Endesfelder, Justine Mondry, Márton Gelléri, Peter J Verveer, and Mike Heilemann. Coordinate-based colocalization analysis of single-molecule localization microscopy data. *Histochemistry and cell biology*, 137(1): 1–10, 2012.

Sebastian Malkusch, Walter Muranyi, Barbara Müller, Hans-Georg Kräusslich, and Mike Heilemann. Single-molecule coordinate-based analysis of the morphology of hiv-1 assembly sites with near-molecular spatial resolution. *Histochemistry and cell biology*, 139(1):173–179, 2013.

Albrecht Ludwig Friedrich Meister. *Generalia de genesi figurarum planarum et inde pendentibus earum affectionibus*. 1769.

Kim I Mortensen, L Stirling Churchman, James A Spudich, and Henrik Flyvbjerg. Optimized localization analysis for single-molecule tracking and super-resolution microscopy. *Nature Methods*, 7(5):377–381, 2010.

Walter Muranyi, Sebastian Malkusch, Barbara Müller, Mike Heilemann, and Hans-Georg Kräusslich. Super-resolution microscopy reveals specific recruitment of hiv-1 envelope proteins to viral assembly sites dependent on the envelope c-terminal tail. *PLoS pathogens*, 9(2):e1003198, 2013.

In Jae Myung. Tutorial on maximum likelihood estimation. *Journal of mathematical Psychology*, 47(1):90–100, 2003.

Robert PJ Nieuwenhuizen, Keith A Lidke, Mark Bates, Daniela Leyton Puig, David Grünwald, Sjoerd Stallinga, and Bernd Rieger. Measuring image resolution in optical nanoscopy. *Nature methods*, 10(6):557–562, 2013.

Martin Ovesnỳ, Pavel Křížek, Josef Borkovec, Zdeněk Švindrych, and Guy M Hagen. Thunderstorm: a comprehensive imagej plug-in for palm and storm data analysis and super-resolution imaging. *Bioinformatics*, 30(16):2389–2390, 2014.

Dylan M Owen, Carles Rentero, Jérémie Rossy, Astrid Magenau, David Williamson, Macarena Rodriguez, and Katharina Gaus. Palm imaging and cluster analysis of protein heterogeneity at the cell surface. *Journal of biophotonics*, 3(7):446–454, 2010.

Sri Rama Prasanna Pavani, Michael A Thompson, Julie S Biteen, Samuel J Lord, Na Liu, Robert J Twieg, Rafael Piestun, and WE Moerner. Three-dimensional, single-molecule fluorescence imaging beyond the diffraction limit by using a double-helix point spread function. *Proceedings of the National Academy of Sciences*, 106(9):2995–2999, 2009.

Thomas Pengo, Seamus J Holden, and Suliana Manley. Palmsiever: a tool to turn raw data into results for single-molecule localization microscopy. *Bioinformatics*, page btu720, 2014.

Bernd Rieger and Sjoerd Stallinga. The lateral and axial localization uncertainty in super-resolution light microscopy. *Chemphyschem*, 15(4):664–670, 2014.

Brian D Ripley. The second-order analysis of stationary point processes. *Journal of Applied Probability*, pages 255–266, 1976.

Michael J Rust, Mark Bates, and Xiaowei Zhuang. Sub-diffraction-limit imaging by stochastic optical reconstruction microscopy (STORM). *Nature Methods*, 3(10): 793–796, 2006.

Daniel Sage, Hagai Kirshner, Thomas Pengo, Nico Stuurman, Junhong Min, Suliana Manley, and Michael Unser. Quantitative evaluation of software packages for single-molecule localization microscopy. *Nature methods*, 2015.

WO Saxton and W_ Baumeister. The correlation averaging of a regularly arranged bacterial cell envelope protein. *Journal of Microscopy*, 127(2):127–138, 1982.

Paul R Selvin, Tyler Lougheed, Melinda Tonks Hoffman, Hyokeun Park, Hamza Balci, Benjamin H Blehm, and Erdal Toprak. Fluorescence Imaging with One-Nanometer Accuracy (FIONA). *Cold Spring Harbor Protocols*, 2007(10), 2007.

Prabuddha Sengupta and Jennifer Lippincott-Schwartz. Quantitative analysis of photoactivated localization microscopy (PALM) datasets using pair-correlation analysis. *Bioessays*, 34(5):396–405, 2012.

Prabuddha Sengupta, Tijana Jovanovic-Talisman, Dunja Skoko, Malte Renz, Sarah L Veatch, and Jennifer Lippincott-Schwartz. Probing protein heterogeneity in the plasma membrane using PALM and pair correlation analysis. *Nature Methods*, 8 (11):969–975, 2011.

Gleb Shtengel, James A Galbraith, Catherine G Galbraith, Jennifer Lippincott-Schwartz, Jennifer M Gillette, Suliana Manley, Rachid Sougrat, Clare M Waterman, Pakorn Kanchanawong, Michael W Davidson, et al. Interferometric fluorescent super-resolution microscopy resolves 3d cellular ultrastructure. *Proceedings of the National Academy of Sciences*, 106(9):3125–3130, 2009.

Sjoerd Stallinga and Bernd Rieger. Accuracy of the gaussian point spread function model in 2d localization microscopy. *Optics express*, 18(24):24461–24476, 2010.

Ernst HK Stelzer. Better imaging through chemistry. *Cell*, 159(6):1243–1246, 2014.

Robert W Sterner, Christine A Ribic, and George E Schatz. Testing for life historical changes in spatial patterns of four tropical tree species. *The Journal of Ecology*, 74 (3):621–633, 1986.

Douglas J Taatjes and Jürgen Roth. The histochemistry and cell biology compendium: a review of 2012. *Histochemistry and cell biology*, 139(6):815–846, 2013.

Russell E Thompson, Daniel R Larson, and Watt W Webb. Precise nanometer localization analysis for individual fluorescent probes. *Biophysical Journal*, 82(5):2775–2783, 2002.

Zinnia Truan, Laura Tarancón Díez, Claudia Bönsch, Sebastian Malkusch, Ulrike Endesfelder, Mihaela Munteanu, Oliver Hartley, Mike Heilemann, and Alexandre Fürstenberg. Quantitative morphological analysis of arrestin2 clustering upon g protein-coupled receptor stimulation by super-resolution microscopy. *Journal of structural biology*, 184(2):329–334, 2013.

Sebastian van de Linde and Markus Sauer. How to switch a fluorophore: from undesired blinking to controlled photoswitching. *Chemical Society Reviews*, 43(4): 1076–1087, 2014.

Steve Wolter, Anna Löschberger, Thorge Holm, Sarah Aufmkolk, Marie-Christine Dabauvalle, Sebastian van de Linde, and Markus Sauer. rapidSTORM: accurate, fast open-source software for localization microscopy. *Nature Methods*, 9(11): 1040–1041, 2012.

Patrick JM Zessin, Carmen L Krüger, Sebastian Malkusch, Ulrike Endesfelder, and Mike Heilemann. A hydrophilic gel matrix for single-molecule super-resolution microscopy. *Optical Nanoscopy*, 2(1):4, 2013.
